# Recent advances in methotrexate pharmacology and potential therapeutic uses in age-related cardiometabolic and neurodegenerative diseases

**DOI:** 10.1016/j.pharmr.2026.100147

**Published:** 2026-06-12

**Authors:** Arduino A. Mangoni, Julie-Ann Hulin, Sara Tommasi

**Affiliations:** 1 Discipline of Clinical Pharmacology, College of Medicine and Public Health, Flinders University, Bedford Park, South Australia, Australia; 2 Flinders Health and Medical Research Institute, College of Medicine and Public Health, Flinders University, Bedford Park, South Australia, Australia; 3 Department of Clinical Pharmacology, Flinders Medical Centre, Southern Adelaide Local Health Network, Bedford Park, South Australia, Australia

## Abstract

Experimental and clinical evidence continues to accumulate, supporting the critical role of dysregulated inflammation, immunity, and redox signaling in the pathophysiology of various age-related cardiometabolic and neurodegenerative diseases. While ongoing research is investigating novel anti-inflammatory and immunomodulatory therapies for such conditions, available antirheumatic drugs may serve a similar purpose. One such drug, methotrexate, has been successfully used at high doses since the 1940s as an anticancer agent and, more recently, at lower doses in patients with autoimmune diseases. Although the effects of methotrexate have traditionally been attributed to its antiproliferative activity via folic acid modulation, additional targets have been identified, including AMP-activated protein kinase, Janus kinase/signal transducer and activator of transcription, high mobility group box 1 protein, the gut microbiota, and additional pharmacological effects of adenosine, a key mediator of methotrexate. The beneficial effects of modulating these targets on downstream inflammatory and immune pathways, cellular senescence, and vascular, metabolic, and brain homeostasis have been increasingly investigated in experimental models. Furthermore, studies conducted over the past 20 years suggest an association between low-dose methotrexate and a decreased risk of certain age-related cardiometabolic and neurodegenerative diseases, particularly in patients with autoimmune conditions. The results of these studies support the potential protective role of methotrexate against age-associated cardiometabolic and neurodegenerative diseases through multiple mechanisms, unlike targeted immunomodulatory and anti-inflammatory drugs, thereby providing a robust framework for investigating its repurposing in future intervention studies.

**Significance Statement:**

New treatments are essential to address the burden of age-related diseases. The important roles of dysregulated inflammation, immunity, and redox signaling in these conditions have spurred research into developing new therapies or repurposing existing drugs to target these dysfunctions. The disease-modifying antirheumatic drug methotrexate has shown potential protective effects against cellular senescence and certain age-related cardiometabolic and neurodegenerative diseases. This knowledge will encourage further research into the repurposing of methotrexate for the treatment of these diseases.

## Graphical abstract

**Figure:**
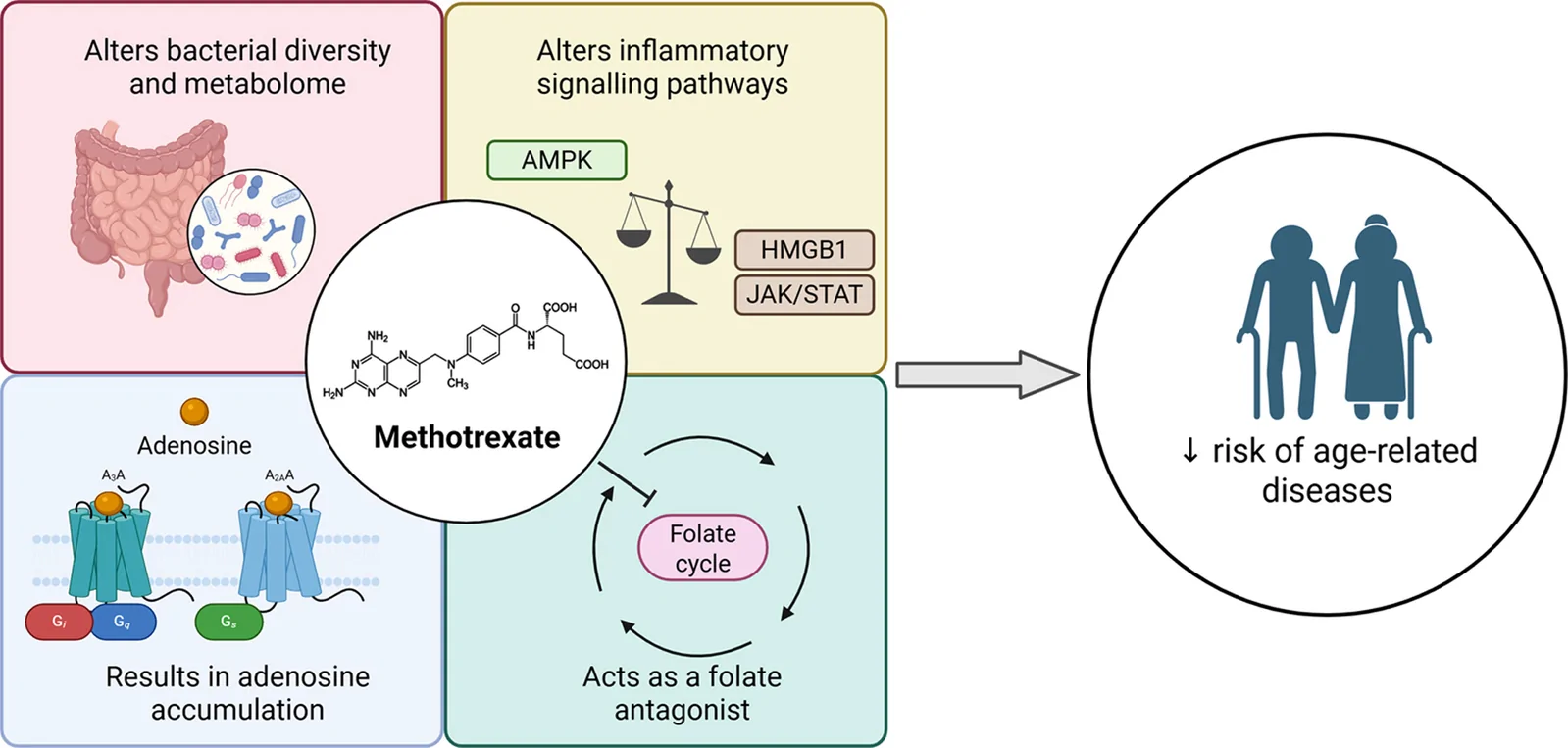

## I Introduction

The gradual aging of the population is associated with higher rates of disease and disability. This, in turn, puts considerable strain on public health and financial resources in healthcare systems worldwide.^1^ A recent systematic review and meta-analysis evaluating multimorbidity, defined as the simultaneous presence of 2 or more chronic health conditions in an individual, found a global prevalence of over 50% among people aged ≥60 years (51.0%; 95% CI, 44.1–58.0).^2^ The link between aging and the risk of disease is well established for many conditions, including cardiometabolic diseases such as atherosclerosis, ischemic heart disease, stroke, and heart failure, along with related risk factors like diabetes mellitus, obesity, insulin resistance, hypertension, dyslipidemia, and metabolic syndrome^3^, ^4^, ^5^; neurodegenerative disorders such as Alzheimer disease, vascular dementia, Lewy body dementia, frontotemporal dementia, and Parkinson disease^6^, ^7^, ^8^, ^9^, ^10^; chronic obstructive pulmonary disease^11^; osteoarthritis^12^; osteoporosis^13^; and specific autoimmune diseases such as rheumatoid arthritis.^14^

A substantial body of research conducted over the past 50 years has revealed several key molecular and cellular changes that underlie the connection between aging and disease states. These changes include genomic instability, telomere dysfunction, epigenetic abnormalities, mitochondrial dysfunction, activation of cellular senescence signaling pathways, abnormal autophagy, loss of proteostasis, and gut microbiota dysbiosis.^15^, ^16^, ^17^, ^18^ At the same time, experimental and clinical evidence continues to grow in support of the idea that a state of chronic, low-grade local and systemic inflammation, often linked to dysregulated immunity and redox signaling, plays a key role in translating these changes into specific disease phenotypes (Fig. 1).^19^ In this context, the concept of “inflammaging,” developed over the past 25 years, has been proposed to describe a state of excessive proinflammatory responses, autoimmunity, and T-cell dysfunction resulting from immunosenescence in adaptive immunity.^20^ “Inflammaging” is also closely linked to altered redox homeostasis, leading to oxidative stress,^21^ recurrent spikes in proinflammatory cytokine secretion,^22^ and dysregulation of specific cellular signaling pathways.^23^ Such pathways include, among others, the nuclear factor *κ*-light-chain enhancer of B cells (NF-*κ*B),^24^ AMP-activated protein kinase (AMPK),^25^ Janus kinase/signal transducer and activator of transcription (JAK/STAT),^26^ high mobility group box 1 protein (HMGB1),^27^ NOD-like receptor protein 3,^28^ mammalian target of rapamycin (mTOR),^29^ transforming growth factor-*β* (TGF-*β*),^30^ tumor necrosis factor-*α* (TNF-*α*),^31^ NOTCH,^32^ and sirtuins (Fig. 1).^33^

**Fig. 1:**
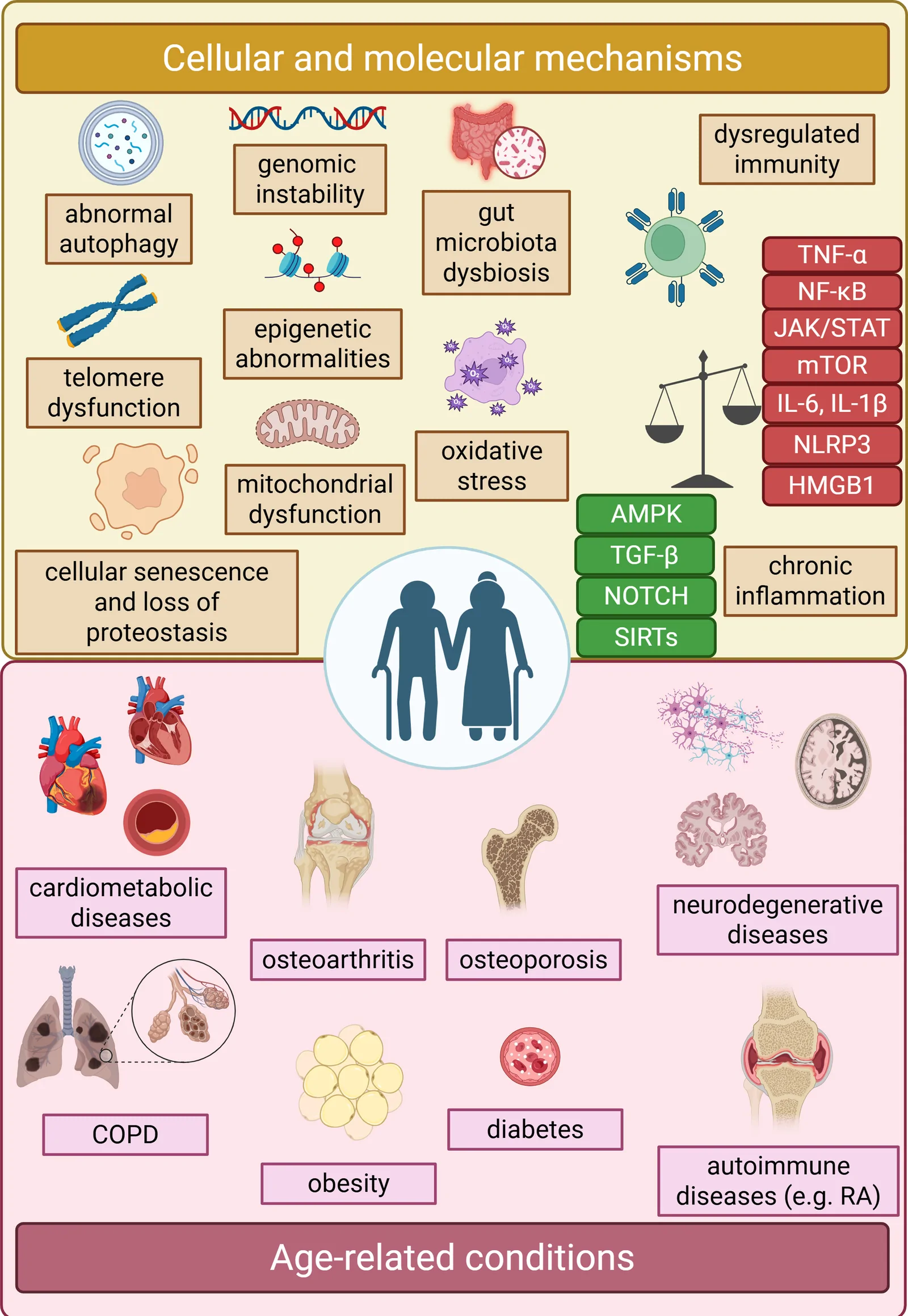
Cellular and molecular mechanisms altered in inflammaging and age-related cardiometabolic and neurodegenerative diseases. Dysregulated inflammation, immunity, redox signaling, genetic and epigenetic abnormalities, and gut microbiome dysbiosis play key roles in the pathophysiology of various age-related cardiometabolic and neurodegenerative diseases. COPD, chronic obstructive pulmonary disease; NLRP3, NOD-like receptor protein 3; RA, rheumatoid arthritis; SIRT, sirtuin.

The central role of inflammation in mediating the complex interplay between aging and disease has significantly influenced drug discovery efforts in academia and industry, with a growing focus on targeting specific inflammatory pathways for prevention and treatment.^34^, ^35^, ^36^, ^37^, ^38^ For example, recognizing that atherosclerosis is mainly caused by a chronic inflammatory state of the arterial wall has led to the development of new treatments targeting specific cytokines, including interleukin (IL)-1, IL-6, IL-2, and IL-17A.^39^ Some of these treatments, such as canakinumab, a monoclonal antibody targeting IL-1*β*, have been shown to reduce cardiovascular morbidity and mortality in intervention studies.^40^ Novel therapeutic strategies targeting inflammation are also being explored for other age-related cardiometabolic and neurodegenerative diseases.^41^, ^42^, ^43^, ^44^, ^45^, ^46^, ^47^, ^48^

An increasingly studied alternative strategy for reducing excessive inflammation in age-related cardiometabolic and neurodegenerative diseases is drug repurposing, also known as drug repositioning, which seeks to identify new uses for existing drugs.^49^ The potential benefits of drug repurposing include reducing manufacturing costs and accelerating early toxicology, preclinical, and phase 1/2 studies, since the main pharmacokinetic properties and toxicity profiles are already known for approved indications.^50^ The extensive clinical experience with repurposed drugs and the resulting availability of robust pharmacoepidemiological safety data constitute a major advantage over new molecules, particularly in patient populations with reduced homeostatic capacity and a higher risk of drug-related toxicity. Furthermore, drug repurposing may help identify new targets and pathways for further investigation using a reverse translation approach.^51^^,^^52^ However, significant limitations of drug repurposing include the lack of commercial incentive after patents expire, complex intellectual property and regulatory pathways for new indications, restricted access to negative or proprietary trial data, and the potential need to re-optimize dose, formulation, and target engagement across tissues. Consequently, late-stage failure and unexpected toxicity remain possible.^49^

One example of a drug successfully repurposed is colchicine. This antimitotic agent, which reduces neutrophil activity and was initially used to treat gout and familial Mediterranean fever, has been shown to lower cardiovascular risk in patients with coronary artery disease.^53^, ^54^, ^55^ Another potential repurposed drug candidate is methotrexate: an antiproliferative, anti-inflammatory, and immunomodulating agent that has been successfully used at high doses since the 1940s as an anticancer drug and, more recently, at low doses as a conventional synthetic disease-modifying antirheumatic drug (csDMARD). Epidemiological studies conducted over the past 20 years on patients with autoimmune disorders, such as rheumatoid arthritis, a model of accelerated inflammaging, suggest an association between low-dose methotrexate and a decreased risk of specific age-related cardiometabolic and neurodegenerative diseases. By contrast, intervention studies failed to demonstrate beneficial cardiometabolic effects of low-dose methotrexate in patients without autoimmune conditions and with a low inflammatory burden. While the therapeutic effects of methotrexate have traditionally been attributed to its antiproliferative activity through modulation of folic acid metabolism, several additional targets have been identified more recently. This knowledge has sparked a growing number of studies exploring the effects of methotrexate and other agents targeting these targets on downstream inflammatory, immune, and redox pathways, as well as on cellular senescence and vascular, metabolic, and brain homeostatic mechanisms. This review article discusses the history and classical pharmacology of methotrexate, with a focus on its anti-inflammatory and immunomodulatory effects; recent molecular targets identified and their pathophysiological relevance; evidence from observational and intervention studies on the association between low-dose methotrexate and the risk of age-related cardiometabolic and neurodegenerative diseases; and possible repurposing applications.

## II The history of methotrexate

The history of methotrexate is intriguing and features early examples of drug repurposing. Methotrexate analogs were first tested in childhood leukemia in 1948 as folic acid antagonists with antiproliferative effects, targeting DNA and RNA synthesis by inhibiting dihydrofolate reductase (DHFR).^56^ Following these observations, methotrexate was successfully used in the 1960s to treat childhood leukemia, with doses of 30 mg/m^2^ administered intramuscularly twice weekly until relapse occurred.^57^ In other malignancies, such as childhood lymphosarcoma and non-Hodgkin’s lymphoma, higher doses of 30 mg/kg given intravenously once a week until toxicity appeared were later studied.^58^

Alongside studies investigating the anticancer effects of methotrexate, trials have also demonstrated its effectiveness in patients with autoimmune diseases. The rationale for repurposing methotrexate to treat these conditions was its antiproliferative effects via DHFR targeting in lymphocytes and other cells involved in intra- and extra-articular inflammatory manifestations. In a double-blind study of patients with psoriatic arthritis, methotrexate was initially administered as a single 25 mg parenteral dose during the first 2 weeks. Afterward, patients receiving active treatment received 3 intravenous or intramuscular injections at 10-day intervals, with doses increasing from 1 to 3 mg/kg.^59^ Subsequent studies successfully investigated lower doses of methotrexate, ranging from 7.5 to 15 mg/wk, in other autoimmune conditions, such as rheumatoid arthritis.^60^^,^^61^ This low-dose treatment approach paved the way for more randomized controlled trials, which confirmed the effectiveness and safety of methotrexate in rheumatoid arthritis and ultimately led to its approval by the US Food and Drug Administration in 1988 for its management.^62^ Subsequent studies have shown that low-dose methotrexate is also effective against other autoimmune diseases, such as systemic lupus erythematosus and systemic sclerosis,^63^^,^^64^ and, more recently, has been used as a first-line treatment for specific conditions involving combined dysregulation of inflammatory and immune pathways, such as sarcoidosis.^65^ Except for high doses for particular types of cancer, such as 4500 mg/m^2^ intravenously and 12 mg intrathecally on days 8, 22, 36, and 50 as part of the BFM 2000 Schema for acute lymphoblastic leukemia,^66^ methotrexate is generally prescribed at relatively low doses ranging from 5 to 30 mg once a week for patients with autoimmune disorders.^67^^,^^68^

## III The pharmacology and pharmacogenetics of methotrexate as an immunomodulator and anti-inflammatory agent

The absorption of oral methotrexate in the gastrointestinal tract is facilitated by solute carrier influx transporters (SLCs). Among these transporters, the proton-coupled folate transporter and the reduced folate carrier are predominantly expressed in the proximal small intestine.^69^, ^70^, ^71^ Although the bioavailability of methotrexate is relatively high, ranging from 64% to 90%, interindividual variability is significant. A decrease in bioavailability is often seen at higher doses, indicating saturation of intestinal transporters.^72^ For these reasons, the weekly administration of methotrexate via the subcutaneous route is becoming increasingly popular in autoimmune disorders, as it lacks a plateau effect on bioavailability, offers higher clinical efficacy, and has an improved safety profile, particularly with respect to gastrointestinal side effects.^73^

The cellular uptake of methotrexate at target sites is primarily mediated by the reduced folate carrier protein 1/SLC19A1 and, to a lesser extent, by the *α* and *β* folate receptors (Fig. 2).^74^ A fundamental shift in understanding the pharmacology of methotrexate occurred following the discovery of the drug’s intracellular accumulation as polyglutamates.^75^ The enzyme folylpolyglutamate synthetase mediates this biotransformation, resulting in the sequential addition of up to 7 glutamate residues to the methotrexate molecule. This process is reversible with the enzyme *γ*-glutamyl hydrolase catalyzing the cleavage of these glutamate residues (Fig. 2).^76^ Methotrexate polyglutamates are considered the pharmacologically active forms of the drug.^76^ Importantly, chains longer than 3 glutamate residues are not substrates for the primary efflux transporters of the ATP-binding cassette family. This increases cellular retention and ensures sustained pharmacological effects, thereby justifying weekly dosing in patients with autoimmune disorders.^77^

**Fig. 2:**
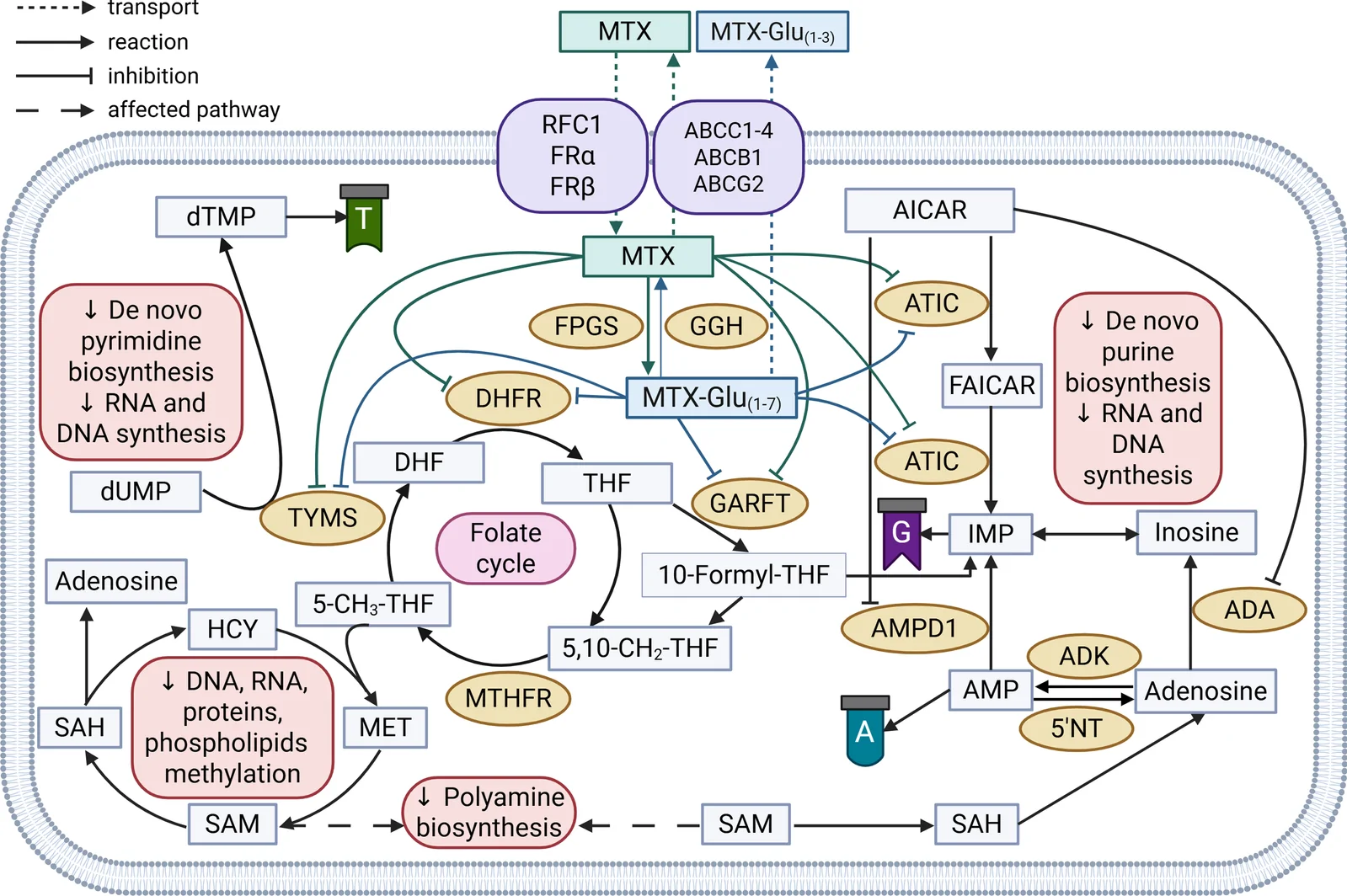
Cellular uptake and mechanisms of methotrexate (MTX) and MTX polyglutamate (MTX-Glu_[n]_) action in the folate cycle and the synthesis of polyamines, purines, and pyrimidines. Following transport-mediated uptake, up to 7 glutamate residues are added to the MTX molecule. Both MTX and its pharmacologically active forms, MTX-Glu_(n)_, interfere with the de novo synthesis of purines and pyrimidines (thus with DNA replication and RNA synthesis), polyamine synthesis, and the methylation of biological molecules by inhibiting multiple enzymes in the folate cycle and the adenosine pathway. Enzymes are encircled in oval boxes, transporters in rounded squares, and metabolites in rectangular boxes. 5,10-CH_2_-THF, 5,10-methylenetetrahydrofolate; 5-CH_3_-THF, 5-methyltetrahydrofolate; 5’NT, 5'-nucleotidase; 10-Formyl-THF, 10-formyltetrahydrofolate; A, adenine; ADK, adenosine kinase; ABCB1, ATP-binding cassette transporter B1; ABCC1-4, ATP-binding cassette transporter 1-4; ABCG2, ATP-binding cassette subfamily G member 2; DHF, dihydrofolate; dTMP, deoxythymidine monophosphate; dUMP, deoxyuridine monophosphate; FAICAR, 5-formamidoimidazole-4-carboxamide ribotide; FPGS, folylpolyglutamate synthetase; FR, folate receptor; G, guanine; GARFT, glycinamide adenine ribonucleotide formyl transferase; GGH, *γ*-glutamyl hydrolase; HCY, homocysteine; IMP, inosine monophosphate; MET, methionine; MTHFR, methylenetetrahydrofolate reductase; RFC1, reduced folate carrier 1; SAH, *S*-adenosylhomocysteine; SAM, *S*-adenosylmethionine; T, thymidine; THF, tetrahydrofolate.

Methotrexate is a folate analog that inhibits DHFR, leading to decreased production of tetrahydrofolate, which acts as a carbon donor in biochemical reactions involved in the synthesis of purines, pyrimidines, and polyamines, as well as in the transmethylation of phospholipids and proteins (Fig. 2).^78^ Both methotrexate and its polyglutamate forms also inhibit other enzymes in the folate pathway, such as thymidylate synthase (TYMS), glycinamide adenine ribonucleotide formyl transferase, and 5-aminoimidazole-4-carboxamide ribonucleotide (AICAR) transformylase/inosine monophosphate cyclohydrolase (ATIC) (Fig. 2). However, the much lower *K*i for DHFR (0.004 nM) in the methotrexate polyglutamate form, MTXglu5, compared with those for TYMS (47 nM), glycinamide adenine ribonucleotide formyl transferase (2500 nM), and ATIC (56 nM) indicates that DHFR is the primary target for folate pathway inhibition.^79^

The decreased synthesis of purines and pyrimidines, and consequently of DNA and RNA, due to targeting DHFR aligns with the antiproliferative effects of high-dose methotrexate in the treatment of malignancies. However, the regular administration of folic acid to reduce toxicity in patients with autoimmune diseases, a widespread practice by clinicians, does not diminish the clinical effectiveness of low-dose methotrexate.^80^ Therefore, while low-dose methotrexate can still exert antiproliferative effects on specific cell types involved in immune activation and tissue damage,^81^ other anti-inflammatory mechanisms of action have been identified. In particular, as a result of ATIC inhibition, the intracellular accumulation of the substrate AICAR leads to the downregulation of 2 critical enzymes that regulate adenosine and AMP metabolism: adenosine deaminase (ADA) and AMP deaminase 1 (AMPD1).^82^ The consequent increase in adenosine concentrations has been shown to mediate the anti-inflammatory effects of methotrexate. In vitro studies have reported that methotrexate increases the release of adenosine from human fibroblasts and endothelial cells.^83^ This, in turn, reduces neutrophil adhesion to fibroblasts. Subsequent experiments in animal models of inflammation have shown that methotrexate-mediated accumulation of adenosine inhibits leukocyte activation and reduces the concentration of the proinflammatory cytokine TNF-*α* (Table 1).^84^^,^^85^

**Table 1:** Biological effects of adenosine that contribute to methotrexate multiorgan protection

Biological Effect	Adenosine Receptor	Reference
Reduction of neutrophil adhesion to fibroblasts	(+) A_2A_	83
Inhibition of leukocyte activation	(+) A_2A_	84
Reduced concentrations of proinflammatory cytokines (eg, TNF-*α*, IL-1*β*, and IL-6) and MMPs	(+) A_2A_/A_3_	85, 86, 87
Inhibition of neutrophil activation, recruitment, and production of superoxide anions	(+) A_2A_	88
Inhibition of osteoclast formation and reduction of bone degradation	(+) A_2A_	89
Transition of macrophages from M1 to M2	(+) A_2A_	89
Inhibition of NF-*κ*B activation in monocytes, macrophages, and endothelial cells	(+) A_2A_	90, 91, 92
Suppression of T-cell activation and apoptosis	(+) A_2A_	77,93
Promotion of the development of T regulatory cells expressing CD39 and CD73	(+) A_2A_	77
Increased expression of cholesterol 27-hydroxylase, ABCA1, and ABCG1, and reduced transformation of THP-1 macrophages into foam cells	(+) A_2A_	94,95
Prevention of COX-2 inhibitor-induced downregulation of ABCA1 and cholesterol 27-hydroxylase and transformation of THP-1 macrophages into foam cells	(+) A_2A_	96
Increased expression of microRNA-181b and inhibition of endothelial cell activation in atherosclerosis and thrombosis	(+) A_3_	97, 98, 99
Reduced concentrations of IL-6 and ICAM-1	(+) A_3_	100
Opening of Kv and KATP potassium channels, vascular smooth cell membrane hyperpolarization, and vasodilation	(+) A_2A_	101
Increased NO production by eNOS	(+) A_2A_	102
Enhanced glucose homeostasis and insulin sensitivity	(+) A_1_/A_2A_/A_2B_	103, 104, 105
Inhibition of lipolysis and reduced circulating concentrations of free fatty acids, triglycerides, and insulin	(+) A_1_	106
Increased insulin-stimulated glucose uptake in skeletal muscle	(+) A_1_	107
Improved glucose regulation and decreased inflammatory markers in blood and adipose tissue	(+) A_2A_	108
Improved motor nerve conduction velocity and nerve blood flow	(+) A_2A_	109
Inhibition of C-Raf/ERK-mediated inflammatory cytokine release and attenuation of retinal cell death associated with diabetes mellitus	(+) A_2A_	110
Reduced urinary albumin excretion, plasma creatinine, inflammatory markers, and nephropathy in animal models of diabetes mellitus	(+) A_2A_	111
Reduction in fatty liver deposition and insulin resistance	(+) A_2A_	112
Prevention of sarcopenia and increased metabolic activity in brown fat	(+) A_2B_	113
Reduction in oxidative stress, lipid peroxidation, and tissue senescence in brown adipose tissue	(+) A_2B_	113
Increased energy expenditure and prevention of diet-induced obesity	(+) A_2B_	113
Increased basal and insulin-stimulated glucose uptake in human myocytes	(+) A_2B_	113
Improved memory, increased IL-10 and PSD-95 concentrations, and decreased TNF-*α* concentrations	(+) A_1_ (−) A_2A_	114
Inhibition of neurotransmission and anxiolytic and anticonvulsant activity	(+) A_1_	86
Neuroprotection and reduction of pain	(+) A_1_/A_3_	86
Stimulation of Glu and ACh release and reduction in locomotory activity	(+) A_2A_	86,115, 116, 117, 118
Excitatory activity, upregulation of proinflammatory markers, and downregulation of anti-inflammatory markers in animal models of stroke	(+) A_2A_	86,119,120

Ach, acetylcholine; COX-2, cycloxygenase-2; C-Raf, C-rapidly accelerated fibrosarcoma serine/threonine kinase; Glu, glutamate; KATP, ATP-sensitive potassium channels; Kv, voltage-gated potassium channels; M1 macrophages, classically activated macrophages; M2 macrophages, alternatively activated macrophages; PSD-95, postsynaptic density protein 95.

Adenosine binds to 4 G protein-coupled receptors: adenosine receptor 1 (A_1_), adenosine receptor 2A (A_2A_), adenosine receptor 2B (A_2B_), and A3, adenosine receptor 3 (A_3_).^86^ The A_1_ and A_3_ receptors are coupled to inhibitory G_i_ proteins and, consequently, downregulate adenylate cyclase activity and reduce intracellular cAMP concentrations.^86^ By contrast, the A_2A_ and A_2B_ receptors are coupled to stimulatory G_s_ proteins and upregulate adenylate cyclase activity, thereby increasing cAMP concentrations (Fig. 3).^86^ Furthermore, adenosine binding to the A_3_ receptor increases the concentrations of Ras homolog family member A (RhoA) and phospholipase D, and activation of the A_3_, A_2A_, and A_2B_ receptors modulates inflammatory, protective, and proliferative cell signaling via p38 mitogen-activated protein kinases, extracellular signal-regulated kinase 1/2 (ERK1/2), and c-Jun N-terminal kinase 1/2 phosphorylation. Adenosine binding to the A_1_, A_3_, and A_2B_ receptors also leads to increased phosphorylation via activation of protein kinase A- and C-dependent signaling cascades (Fig. 3).^86^ The available evidence suggests that the anti-inflammatory effects, such as decreased production of TNF-*α*, IL-1*β*, IL-6, and matrix metalloproteinases (MMPs), are primarily mediated by activation of the A_2A_ and A_3_ receptors (Fig. 3; Table 1).^86^^,^^87^ Adenosine signaling through A_2A_ receptor activation has also been shown to inhibit the production of superoxide anions by neutrophils, as well as their adhesion and recruitment.^88^ Additionally, it inhibits osteoclast formation, leading to reduced bone degradation, promotes the transition of macrophages from the M1 (proinflammatory) to the M2 (anti-inflammatory) type,^89^ and inhibits NF-*κ*B activation in monocytes, macrophages, and endothelial cells.^90^, ^91^, ^92^ A_2A_ receptor-mediated signaling has also been shown to suppress T-cell activation and promote the development of T regulatory cells expressing CD39 and CD73, which further decrease T-cell activation (Fig. 3; Table 1).^77^^,^^93^

**Fig. 3:**
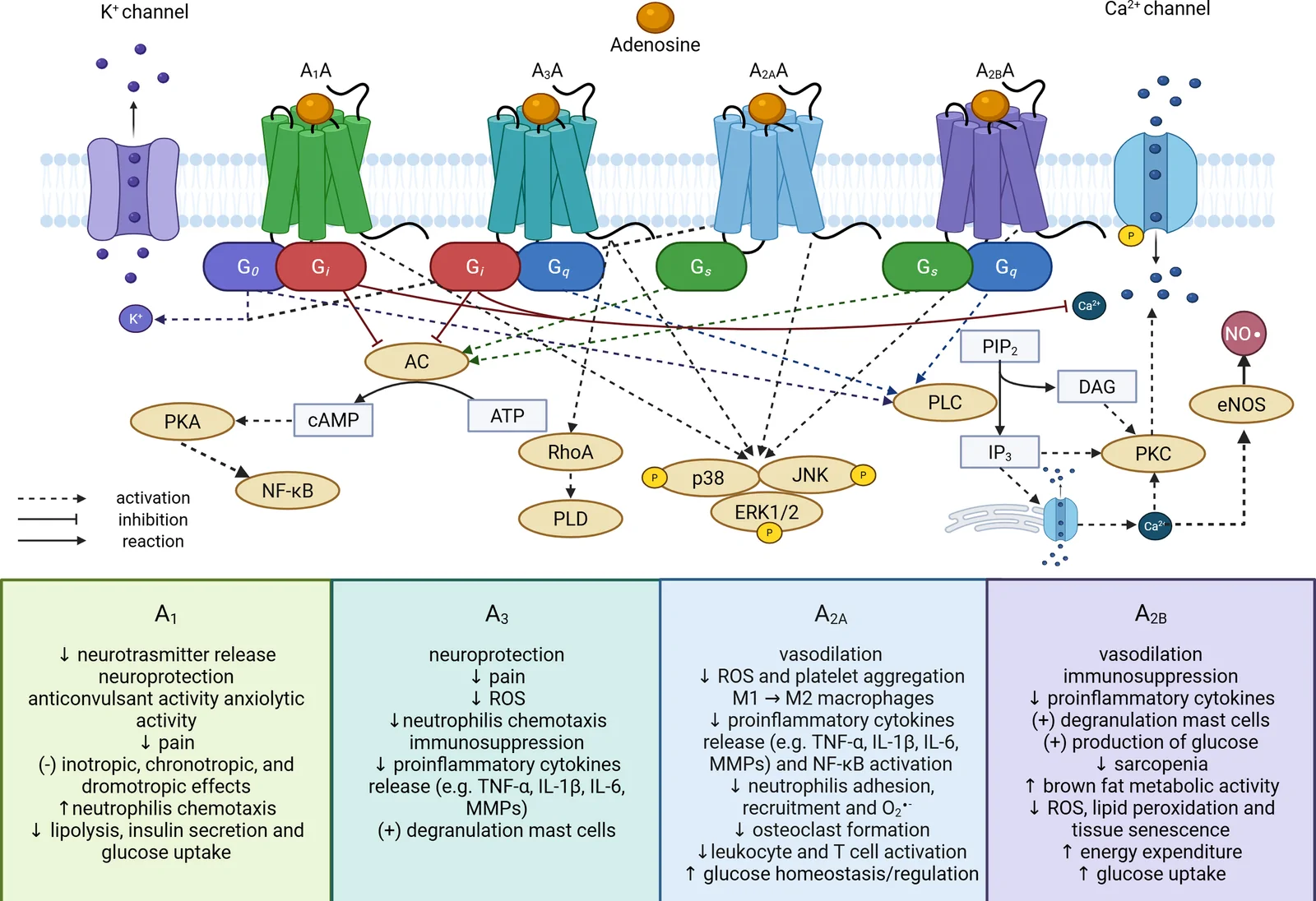
Anti-inflammatory, cardioprotective, and neuroprotective effects of adenosine. Adenosine binds to 4 G protein-coupled receptors: A_1_, A_2A_, A_2B_, and A_3_. The A_1_ and A_3_ receptors are coupled to the inhibitory G_i_ proteins and the G_*0*_ and G*q* proteins, respectively. Binding to these receptors downregulates adenylate cyclase (AC) and reduces intracellular cAMP concentrations, while favoring potassium (K^+^) efflux and increasing intracellular calcium (Ca^2+^) concentrations. By contrast, the A_2A_ and A_2B_ receptors are coupled to stimulatory G_s_ proteins and upregulate AC, thereby increasing cAMP concentrations. The binding of adenosine to the A_2A_ receptor also activates a signaling cascade mediated by the mitogen-activated protein kinases p38 (p38), c-Jun N-terminal kinase (JNK), and ERK1/2, whereas binding to the A_2B_ receptor is associated with increased intracellular Ca^2+^ concentrations and kinase activation via the inositol trisphosphate (IP_3_)/diacylglycerol (DAG) pathway. M1 macrophages, classically activated macrophages; M2 macrophages, alternatively activated macrophages; O_2_^∙-^, superoxide anion; P, phosphate; PIP_2_, phosphatidylinositol 4,5-bisphosphate; PKA, protein kinase A; PKC, protein kinase C; PLC, phospholipase C; PLD, phospholipase D; RhoA, Ras homolog family member A; ROS, reactive oxygen species.

The relative contribution of the A_2A_ and A_3_ receptors to the anti-inflammatory effects of methotrexate in vivo may depend on the specific experimental model and/or the tissue pathway used. For example, in the murine air-pouch model, low-dose methotrexate increased adenosine concentrations in the exudates and reduced leukocyte accumulation and TNF-*α* concentrations in the exudates of wild-type mice, but not in those of A_2A_ or A_3_ receptor knockout mice.^85^ By contrast, in a model of thioglycolate-induced peritonitis, suppression of inflammation by low-dose methotrexate was mediated by A_2A_ receptors but not by A_3_ receptors.^121^ Given that the A_3_ receptor couples primarily to Gi (lowering cAMP) but can signal via mitogen-activated protein kinases, phosphoinositide 3-kinases, and NF-*κ*B modulatory pathways (Fig. 3), cAMP elevation should be considered a central effector of A_2A_ receptors, with A_3_-dependent, cAMP-independent signaling potentially acting as an ancillary mechanism in selected inflammatory conditions.^86^ Therefore, the anti-inflammatory effects of low-dose methotrexate, including folic acid modulation, cAMP elevation, and cAMP-independent signaling, differ from those of other drugs, eg, phosphodiesterase inhibitors, such as apremilast, act downstream by blocking cAMP hydrolysis, amplifying the protein kinase A/cAMP response element-binding protein and exchange protein directly activated by cAMP pathways, and suppressing NF-*κ*B–dependent pathways independent of adenosine generation.^122^

In summary, although DHFR inhibition contributes to antiproliferative effects, the immunomodulatory effects of methotrexate are largely attributed to ATIC inhibition, which leads to AICAR accumulation and consequent downregulation of ADA and AMPD1, thereby increasing extracellular adenosine. Adenosine signaling, predominantly via the A_2A_ and A_3_ receptors, reduces leukocyte adhesion, oxidative burst, and recruitment; suppresses NF-*κ*B–dependent inflammatory pathways; decreases production of TNF-*α*, IL-1*β*, IL-6, and MMPs; promotes M2 macrophage polarization; and enhances regulatory T-cell pathways.

Given the complex mechanisms regulating methotrexate cellular uptake and retention, as well as its target enzymes, several studies have examined pharmacogenetic biomarkers of low-dose treatment response in autoimmune and inflammatory conditions, particularly rheumatoid arthritis.^123^ A systematic review has critically appraised the existing evidence on single-nucleotide polymorphisms (SNPs) that regulate transporters, polyglutamates, adenosine, and folic acid pathways, as well as purines, pyrimidines, methionine, and ubiquitin.^124^ An analysis of 35 articles and 7 meta-analyses led to the identification of 6 SNPs (after Bonferroni correction) consistently associated with methotrexate efficacy: *ATIC* rs7563206, *SLC19A1* rs1051266, *DHFR* rs836788, and *TYMS* rs2244500, rs2847153, and rs3786362. *ATIC* rs7563206, *TYMS* rs2847153, and *TYMS* rs3786362 were associated with a lack of response, whereas *SLC19A1* rs1051266, *DHFR* rs836788, and *TYMS* rs2244500 were associated with treatment response. Of the selected SNPs, *SLC19A1* rs1051266 had the most robust supporting evidence, with 2 studies showing significant associations with methotrexate response. Other SNPs either showed associations with methotrexate efficacy in individual studies or exhibited inconsistent results: *AMPD1* rs17602729, *ATIC* rs2372536, *ATIC* rs4673993, *ATIC* rs12995526, *FPGS* rs4451422, methylenetetrahydrofolate reductase (*MTHFR*) rs1801133, methionine synthase (*MTR*) rs1805087, and cullin 1 (*CUL1*) rs122571. A subsequent systematic review and meta-analysis of *ATIC* rs2372536 across 13 studies involving 3185 patients with rheumatoid arthritis showed a significant association with a lack of response, particularly in European populations (odds ratio [OR], 0.548; 95% CI, 0.591–0.929).^125^

## IV Emerging targets of methotrexate and potential therapeutic uses in age-related cardiometabolic and neurodegenerative diseases

The following sections outline recent advances in identifying novel methotrexate targets for age-related cardiometabolic and neurodegenerative diseases.

### A Additional pharmacological effects of adenosine

A_2A_ receptor activation increases the expression of cholesterol 27-hydroxylase, ATP-binding cassette transporter A1 (ABCA1), and ATP-binding cassette subfamily G member 1 (ABCG1) in stimulated THP-1 human macrophages (Table 1).^94^^,^^95^ This enhances cholesterol efflux, thereby attenuating cholesterol accumulation in monocyte-derived macrophages within the arterial wall, a crucial step in atherosclerosis.^95^^,^^96^^,^^126^, ^127^, ^128^ Furthermore, methotrexate-mediated activation of the A_3_ receptor increases the expression of microRNA-181b, an anti-inflammatory microRNA that inhibits endothelial activation in atherosclerosis and thrombosis, at concentrations of 1–100 *μ*M (Table 1).^97^, ^98^, ^99^ The effects observed at 1 *μ*M methotrexate may be biologically and clinically relevant, as this concentration is similar to that measured in the serum of rheumatoid arthritis patients treated with low-dose methotrexate.^129^

Adenosine relaxes vascular smooth muscle, thereby reducing peripheral vascular resistance and increasing blood flow (Fig. 3; Table 1). This effect, extensively studied in the coronary circulation, is mediated by activation of the A_2A_ receptor.^101^ A_2A_ receptor activation in endothelial cells also stimulates nitric oxide (NO) production by endothelial NO synthase (eNOS).^102^

Activation of adenosine receptors enhances glucose homeostasis and insulin sensitivity (Fig. 3; Table 1). Partial A_1_ agonists reduced circulating insulin in rats fed a high-fat diet,^111^ whereas A_1_ agonists increased insulin-stimulated glucose uptake in skeletal muscle.^107^ Selective A_2A_ agonists improved glucose regulation in rats fed a high-fat diet through anti-inflammatory effects.^103^^,^^108^ The A_2B_ receptor also plays a key role in the interplay among adipose tissue, skeletal muscle, and aging (Fig. 3; Table 1). A_2B_ agonists reduce senescence in brown adipose tissue, increase energy expenditure, and improve glucose tolerance in mice.^113^

A_2A_ inhibition exerts neuroprotective effects by enhancing autophagy, antioxidant effects, and synaptic function in mice.^130^^,^^131^ Combined treatment with A_1_ agonists and A_2A_ antagonists improves memory in Alzheimer disease models (Table 1).^114^ A_2A_ antagonists have also been associated with preserved memory and cognitive function in mice.^132^ The predominant localization of A_2A_ receptors in the basal ganglia and limbic system, and their ability to regulate numerous neurotransmitters, also offer potential therapeutic opportunities in Parkinson disease.^115^ A_2A_ antagonists reverse motor impairment and improve behavioral domains in animal models and in patients with Parkinson disease and in rat models of small-vessel stroke.^116^, ^117^, ^118^, ^119^, ^120^

Although dysregulated inflammation is central to age-related cardiometabolic and neurodegenerative diseases, several protective effects of adenosine are at least partly independent of inflammatory modulation. These include promotion of cholesterol efflux and reduced foam cell formation; inhibition of restenosis-like responses; endothelial vasomotor support and flow preservation (including regulation of cerebral blood flow); reduction in circulating free fatty acids; insulin-mediated skeletal muscle glucose transport; and improved synaptic function.

### B AMP-activated protein kinase activation

As previously described, methotrexate-induced inhibition of ATIC causes AICAR accumulation (Figs. 2 and 4).^82^ In addition to downregulating ADA and AMPD1, AICAR activates AMPK,^133^ a critical regulator of cellular metabolism and energy balance.^134^ The AICAR precursor, 5-amino-4-imidazolecarboxamide ribonucleoside, enters the cell via adenosine transporters and is converted into its monophosphorylated derivative (5-aminoimidazole-4-carboxamide ribonucleotide or AICAR) by the enzyme adenosine kinase.^135^ AICAR then acts as an AMP mimetic that binds to cystathionine-*ß*-synthase sites on the AMPK *γ*-subunit within the *αβγ* heterotrimer, promoting Thr172 phosphorylation and allosteric activation.^136^^,^^137^ Because *γ*1-containing complexes account for most AMPK activity across tissues, AICAR is expected to activate both *α*1- and *α*2-based heterotrimers predominantly through *γ*1.^137^ In glycolytic skeletal muscle, however, AICAR-stimulated activity is largely attributable to *α*2 complexes and is enriched in *α*2*β*2*γ*3 assemblies, reflecting isoform distribution rather than strict AICAR selectivity.^138^ There is growing evidence that AMPK activation benefits cardiometabolic and neurodegenerative pathologies.^139^, ^140^, ^141^, ^142^, ^143^

**Fig. 4:**
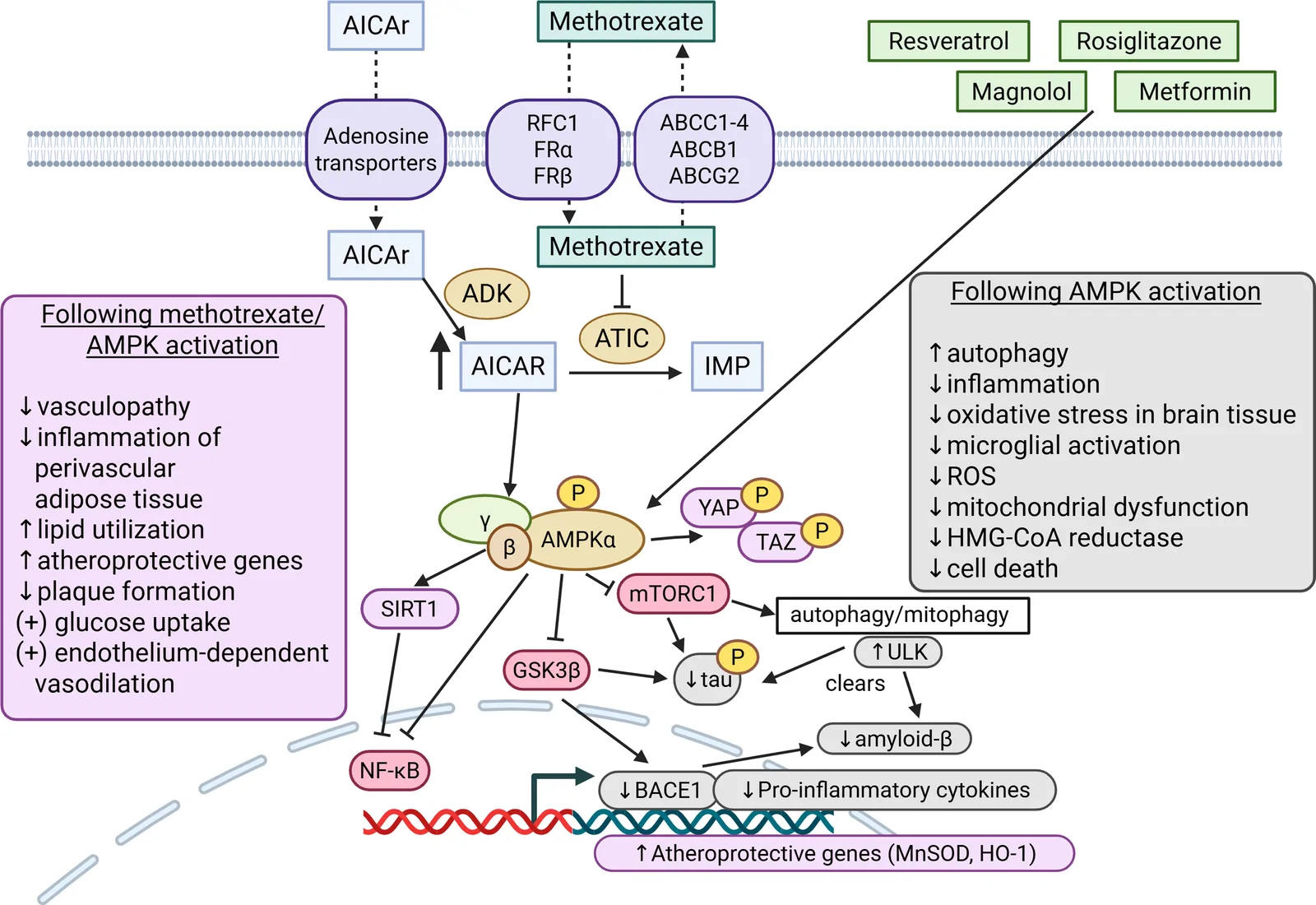
Effect of AMPK activation on age-related cardiometabolic and neurodegenerative diseases. Resveratrol, rosiglitazone, magnolol, and metformin are AMPK activators. Methotrexate-induced inhibition of ATIC results in the accumulation of the substrate AICAR, which also activates AMPK. Activation of AMPK results in the downregulation of proinflammatory cytokines and the upregulation of atheroprotective genes, ultimately leading to positive effects on cardiometabolic and neurodegenerative pathologies. Outcomes in the purple box are effects observed in models of cardiometabolic disease; outcomes in the gray box support a protective role for AMPK in neurodegenerative disorders. ABCB1, ATP-binding cassette transporter B1; ABCC1-4, ATP-binding cassette transporter 1-4; ABCG2, ATP-binding cassette subfamily G member 2; ADK, adenosine kinase; AICAr, 5-aminoimidazole-4-carboxamide ribonucleoside; FR, folate receptor; GSK3*β*, glycogen synthase kinase-3*β*; HO-1, heme oxygenase-1; IMP, inosine monophosphate; MnSOD, manganese superoxide dismutase; P, phosphate; RFC1, reduced folate carrier 1; ROS, reactive oxygen species; SIRT1, sirtuin 1; TAZ, transcriptional coactivator with PDZ-binding motif; ULK, Unc-51-like kinase; YAP, yes-associated protein.

Several studies report the protective effects of methotrexate against cardiometabolic diseases through activation of AMPK (Fig. 4). Methotrexate-induced AMPK activation reduces vasculopathy and plaque formation, increases the expression of atheroprotective genes, and improves endothelium-dependent vasodilation at concentrations similar to those measured in rheumatoid arthritis patients treated with low-dose methotrexate.^129^^,^^144^, ^145^, ^146^, ^147^ AMPK activation is also known to inhibit 3-hydroxy-3-methylglutaryl-coenzyme A (HMG-CoA) reductase, thereby reducing cholesterol synthesis and enhancing lipid homeostasis by suppressing lipogenesis and promoting lipid disposal.^148^, ^149^, ^150^ In cultured myotubes, methotrexate lowers the threshold for AICAR-induced AMPK activation, increases lipid utilization, and stimulates glucose uptake.^104^ In diabetic mice, low-dose methotrexate increased skeletal muscle glucose transporter type 4 (GLUT4) expression and reduced glucose and insulin concentrations.^105^ In another study of diabetic mice, a 2-week treatment with higher doses of methotrexate (2–4 mg/kg weekly) exerted significant anti-inflammatory and lipolytic effects in visceral adipose tissue.^151^

Growing evidence also supports a potential protective role for AMPK activation in neurodegenerative disorders (Fig. 4). AMPK stimulation reduces amyloid-*β* generation and deposition by decreasing *β*-site amyloid precursor protein-cleaving enzyme 1 (BACE1) abundance through regulation of BACE1 translation-associated ribonucleoprotein complexes and by suppressing mTOR, glycogen synthase kinase 3*β*, and NF-*κ*B signaling.^152^, ^153^, ^154^, ^155^, ^156^ AMPK also inhibits mTOR complex 1 (mTORC1) activity through tuberous sclerosis complex 2 and raptor-dependent mechanisms, thereby reducing tau phosphorylation and aggregation while increasing Unc-51-like kinase activity, autophagy, and mitophagy, thereby enhancing clearance of aggregation-prone proteins.^142^^,^^157^ Furthermore, the interaction between AMPK and the silent information regulator sirtuin 1 suppresses NF-*κ*B transcriptional activity, thereby attenuating neuroinflammation and oxidative injury.^141^^,^^158^^,^^159^ Several AMPK activators have improved cognitive function in animal and clinical studies.^160^, ^161^, ^162^, ^163^, ^164^ Furthermore, AMPK overexpression reduced cell death and exerted protective effects in experimental models of Parkinson disease.^165^^,^^166^

### C AMP-activated protein kinase-independent effects of 5-aminoimidazole-4-carboxamide ribonucleotide

AICAR also exerts direct protective effects against cardiometabolic and neurodegenerative pathologies independently of AMPK (Fig. 5). In hepatocytes, AICAR increases low-density lipoprotein (LDL) receptor mRNA expression and protein levels through ERK1/2.^167^ AICAR also suppresses peroxisome proliferator-activated receptor *γ* (PPAR*γ*) expression and PPAR*γ* target gene activation, which are critical for oxidized LDL uptake and an increased inflammatory response.^168^ These effects are not influenced by AMPK inhibitors or *AMPK* silencing. Furthermore, AICAR attenuates the endoplasmic reticulum stress response, a key pathophysiological step in type 2 diabetes.^169^ These effects persist in the presence of AMPK inhibitors or *AMPK* silencing.

**Fig. 5:**
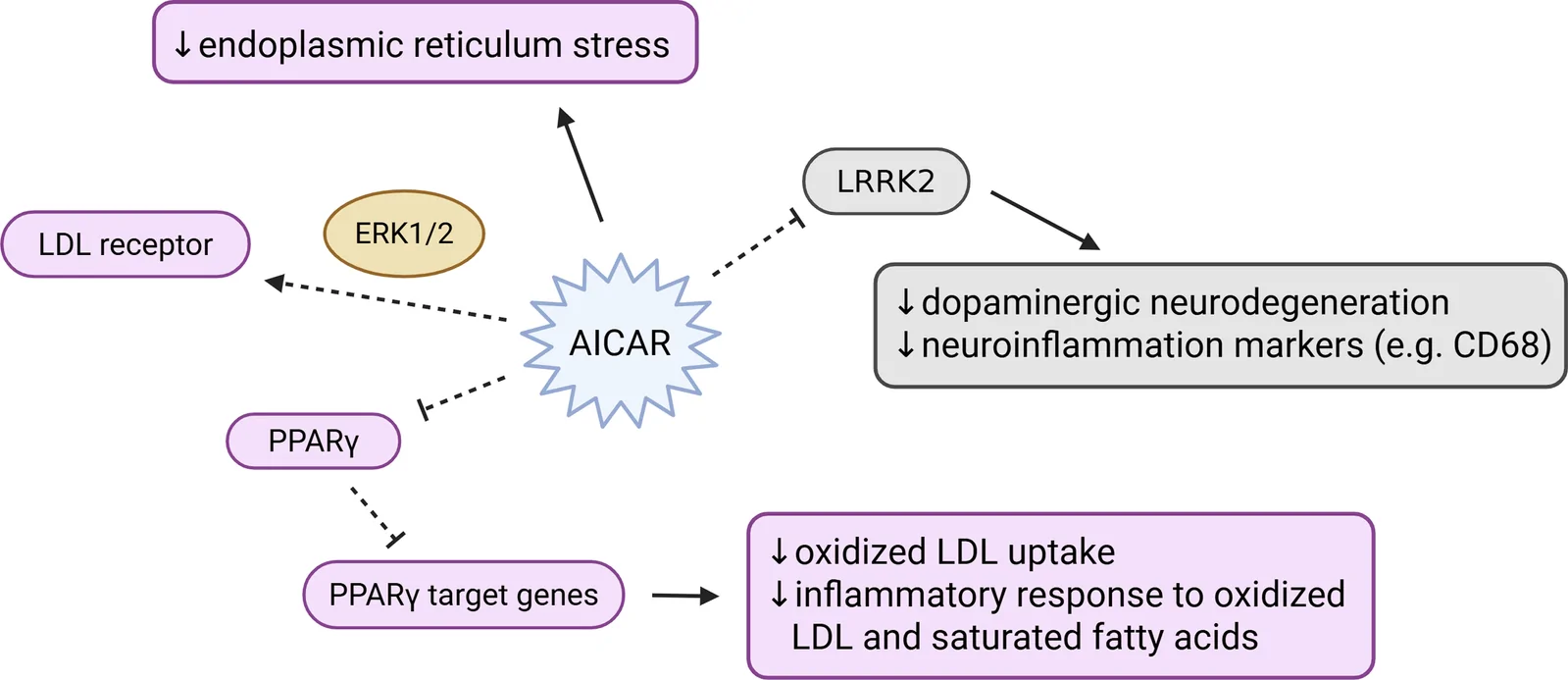
Independent effects of AICAR on age-related cardiometabolic and neurodegenerative diseases. AICAR has been shown to provide direct protective effects against age-related cardiometabolic and neurodegenerative pathologies. Protective effects include reduced endoplasmic reticulum stress, reduced uptake of oxidized LDL, and decreased dopaminergic neurodegeneration. Purple boxes indicate cardiometabolic-related effects; gray boxes indicate neurodegenerative-related effects. CD68, cluster of differentiation 68.

AICAR also downregulates leucine-rich repeat kinase 2 (LRRK2) expression in experimental models of Parkinson disease, conferring protection against dopaminergic neurodegeneration and reducing neuroinflammatory markers.^170^

### D Janus kinase/signal transducer and activator of transcription pathway inhibition

The JAK/STAT signaling pathway, discovered in 1990,^171^ is a highly conserved pathway that regulates numerous cellular processes, including inflammatory mediator production, through the activation of specific transmembrane receptors, such as type I and type II cytokine receptors.^26^ These receptors bind several cytokines, including IL-6, IL-10, and IL-12, as well as interferons (IFNs) and hormones.^172^ Mammals express 4 Janus kinase (JAK) family members: JAK1, JAK2, JAK3, and tyrosine kinase 2, each with distinct receptor selectivity and a similar architecture consisting of an N-terminal interaction domain, a Src-homology 2 domain, a central pseudo-kinase regulatory domain, and a C-terminal tyrosine kinase domain.^26^ Cytokine binding activates JAKs, leading to signal transducer and activator of transcription (STAT) docking, activation, phosphorylation, and nuclear translocation of STAT1, STAT2, STAT3, STAT4, STAT5a, STAT5b, and STAT6, thereby activating or suppressing target genes.^26^^,^^173^

Excessive JAK/STAT activation plays a critical role in cancer, autoimmune diseases, and, more broadly, age-related diseases and cellular aging.^26^ There is also growing evidence that JAK/STAT upregulation promotes atherosclerosis,^174^ obesity, diabetes mellitus, metabolic syndrome,^175^^,^^176^ Alzheimer disease,^177^ and Parkinson disease (Fig. 6).^178^

**Fig. 6:**
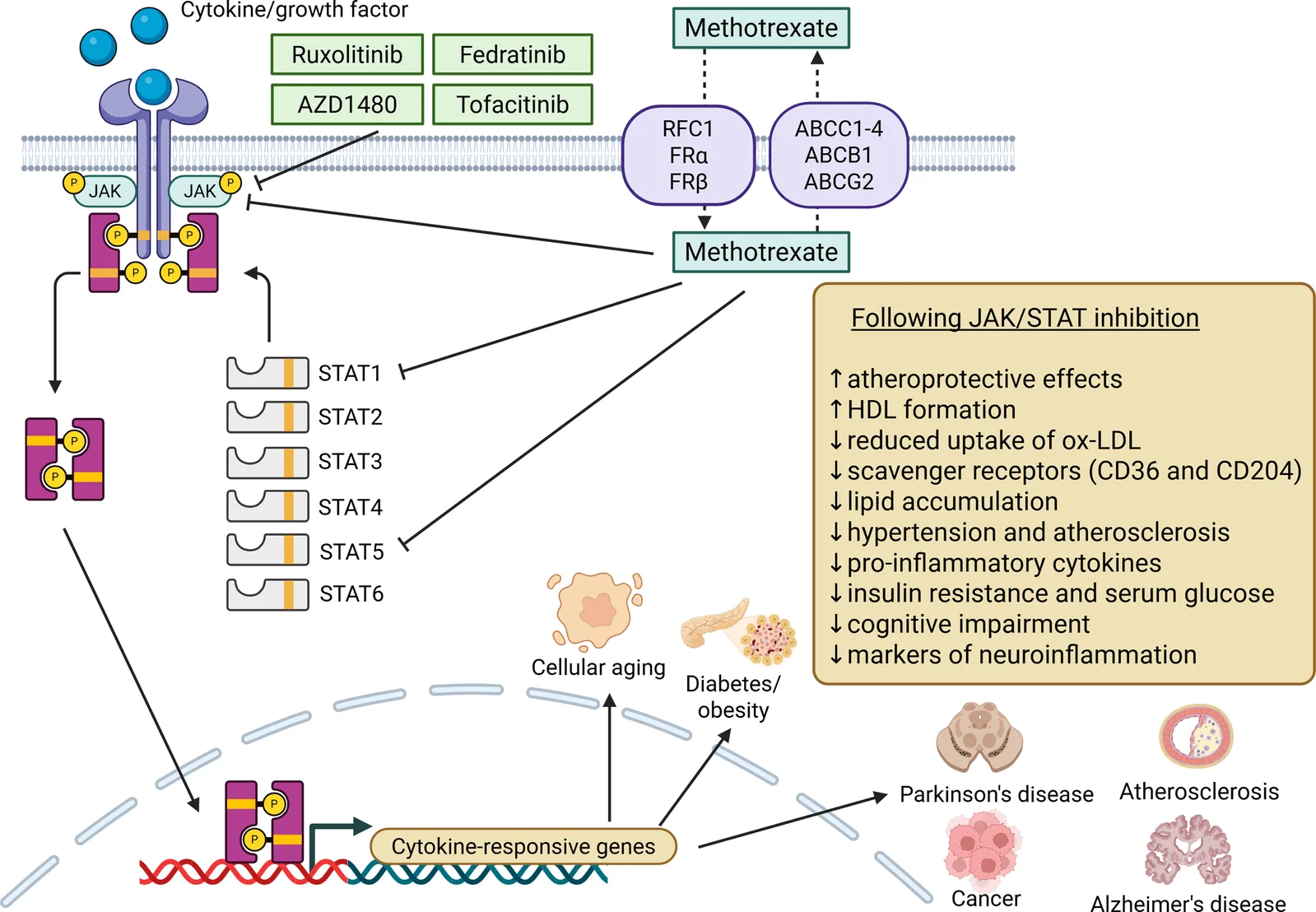
Effect of JAK/STAT inhibition on age-related cardiometabolic and neurodegenerative diseases. The JAK/STAT pathway is activated by the binding of cytokines to their receptors. The associated JAK proteins transphosphorylate and activate one another, creating docking sites for STAT proteins and leading to their phosphorylation and activation. Excess JAK/STAT activation can lead to cancer, cellular aging, and age-related cardiometabolic and neurodegenerative diseases. Inhibition of JAK/STAT signaling by ruxolitinib, fedratinib, and tofacitinib can reduce lipid accumulation, enhance atheroprotective effects, and reduce cognitive impairment, among other effects. Methotrexate also inhibits the JAK/STAT pathway via inhibition of both JAK and STAT protein phosphorylation. ABCB1, ATP-binding cassette transporter B1; ABCC1-4, ATP-binding cassette transporter 1-4; ABCG2, ATP-binding cassette subfamily G member 2; FR, folate receptor; ox-LDL, oxidized LDL; P, phosphate; RFC1, reduced folate carrier 1.

Studies over the last decade indicate that methotrexate significantly inhibits the JAK/STAT pathway. Screening of a 2000 compound small-molecule library first identified methotrexate and aminopterin as potent JAK/STAT inhibitors in *Drosophila*.^179^ In human erythroid leukemia-derived cells, both drugs decreased constitutive phosphorylation of JAK1 and, to a lesser extent, JAK2, and reduced phosphorylation of STAT1 and STAT5, but not STAT3, in a dose-dependent manner. Suppression of STAT5 phosphorylation occurred at methotrexate concentrations (1 *μ*M) similar to those measured in patients receiving low-dose treatment and was comparable to that observed with the known JAK1/2 inhibitor ruxolitinib.^129^^,^^179^ Methotrexate also inhibits STAT phosphorylation in animal models of myeloproliferative disorders, consistent with human studies showing beneficial effects of low-dose methotrexate on clinical activity markers in patients with myeloproliferative disorders and JAK2 V617F mutations.^180^, ^181^, ^182^ In silico docking studies suggest that methotrexate can occupy the JAK2 ATP-binding pocket with greater affinity than ATP, indicating competitive kinase inhibition.^180^ Transcriptomic analysis of CD4+ cells from patients with rheumatoid arthritis shows that p53 and JAK/STAT signaling are the only pathways affected by methotrexate.^183^

Although not studied explicitly with methotrexate, emerging evidence suggests that JAK/STAT inhibition may be a promising therapeutic strategy in age-related cardiometabolic and neurodegenerative diseases (Fig. 6). Both ruxolitinib and tofacitinib, JAK1/3 inhibitors, reduce lipid accumulation in macrophages, a critical step in atherogenesis.^184^, ^185^, ^186^ JAK/STAT inhibition also prevents excessive production of the vasoconstrictor endothelin-1 in endothelial cells exposed to high glucose.^187^^,^^188^ Atheroprotective effects have also been reported in animal studies with the JAK2 inhibitor fedratinib and with ruxolitinib.^189^^,^^190^ In animal models of type 2 diabetes, tofacitinib reduces proinflammatory cytokines, insulin resistance, and serum glucose levels, while increasing GLUT4 expression.^191^ In patients with rheumatoid arthritis, with or without type 2 diabetes, tofacitinib and baricitinib reduce insulin resistance and glycated hemoglobin.^192^, ^193^, ^194^ JAK/STAT inhibition has also shown beneficial effects in diabetic nephropathy.^195^

Some studies also report promising effects of JAK/STAT inhibition in experimental models of cerebral ischemia-reperfusion injury, human immunodeficiency virus-related neurocognitive disorder, and Parkinson disease. These effects are associated with reduced anxiety-like behavior and hippocampal neuronal loss, antioxidant and anti-inflammatory effects, and suppression of microglial activation and cell apoptotic pathways.^196^, ^197^, ^198^, ^199^

While the available evidence supports the beneficial effects of targeting JAK/STAT signaling in several age-related cardiometabolic and neurodegenerative diseases, concerns have been raised about the possible cardiovascular toxicity of certain JAK/STAT inhibitors in rheumatoid arthritis, such as tofacitinib.^200^ However, recent systematic reviews and meta-analyses have not reported a significant increase in cardiovascular risk with various JAK/STAT inhibitors, including tofacitinib, across a wide range of autoimmune disorders.^201^^,^^202^

### E High mobility group box 1 protein inhibition

HMGB1, discovered in 1973,^203^ is a highly conserved protein found in the nucleus, cytoplasm, and extracellular space.^204^ Early research focused on nuclear HMGB1 and its role in maintaining DNA structure and preventing DNA damage. Later studies identified extranuclear HMGB1 as a key mediator of immune activation through its interactions with several receptors, particularly toll-like receptor (TLR) 4 and the advanced glycosylation end product-specific receptor (AGER). Activation of these receptors ultimately triggers NF-*κ*B and IFN regulatory factor 3 signaling, leading to the production of multiple cytokines and chemokines.^204^ HMGB1 activation therefore contributes to infection, inflammation, autoimmune conditions, cancer, cellular senescence, and age-related cardiometabolic and neurodegenerative diseases (Fig. 7).^204^ Aging cells develop a senescence-associated secretory phenotype that includes HMGB1 release, and redox-sensitive HMGB1 promotes senescence-like phenotypes through JAK/STAT and NF-*κ*B signaling, whereas HMGB1 inhibition prevents them.^205^ Excessive HMGB1 release may also be triggered by an age-related decline in HMGB1 expression, leading to DNA and cellular damage and contributing to age-related neuroinflammation.^206^^,^^207^ HMGB1 also promotes amyloid-*β* accumulation and cognitive impairment.^208^

**Fig. 7:**
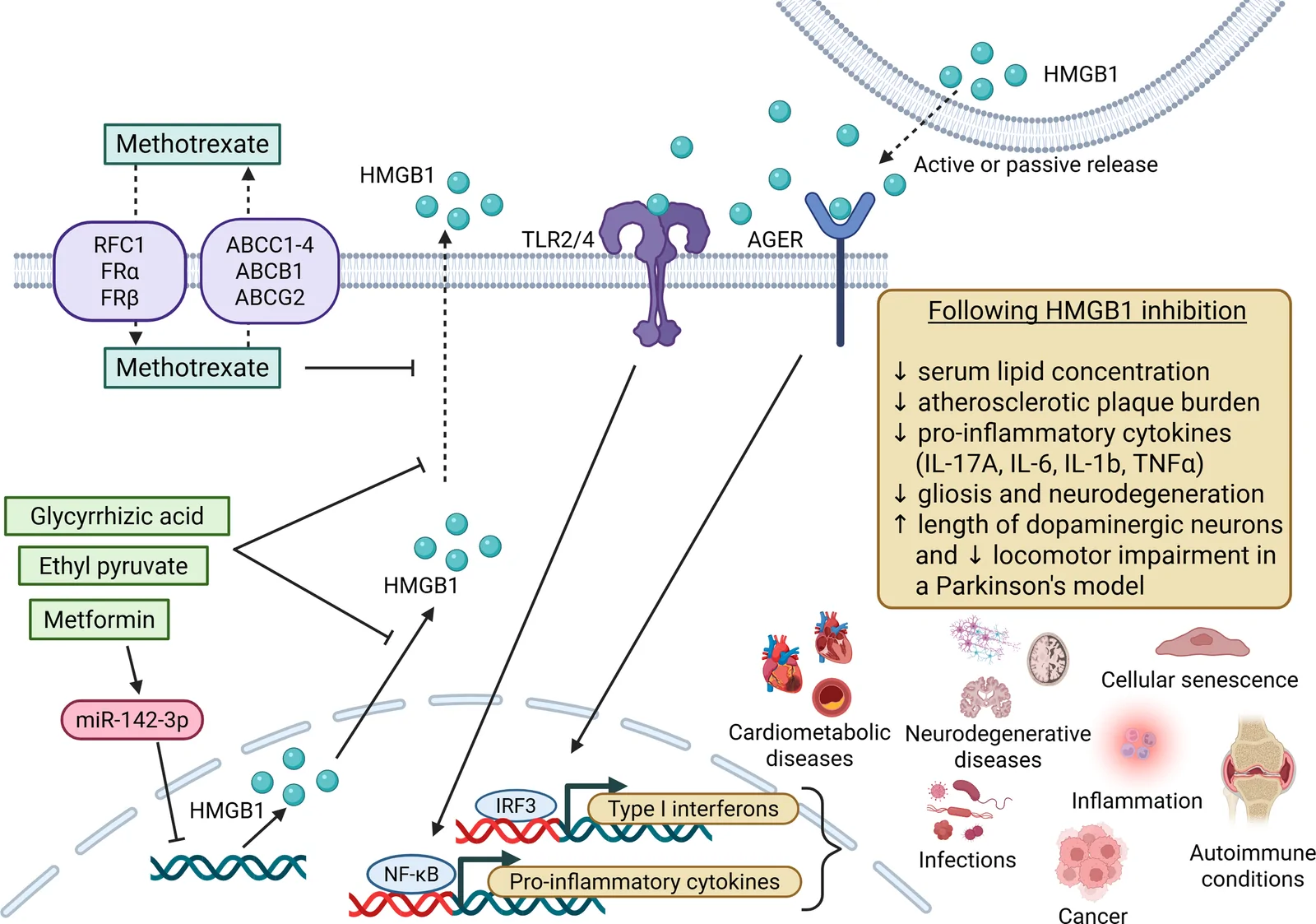
Effect of HMGB1 inhibition on age-related cardiometabolic and neurodegenerative diseases. HMGB1 is released from cells via passive release following cell death or via active secretion by immune cells in response to inflammation or infection. HMGB1 binds to TLR2/4 or AGER receptors, initiating downstream signaling pathways that ultimately activate NF-*κ*B and IFN regulatory factor 3. This leads to the production of multiple cytokines and chemokines, ultimately resulting in cellular senescence, cancer, infection, and inflammation. Inhibition of HMGB1 signaling has been shown to reduce serum lipid concentrations, decrease proinflammatory cytokines, and increase the length of dopaminergic neurons. Methotrexate has been reported to inhibit HMGB1 signaling. ABCB1, ATP-binding cassette transporter B1; ABCC1-4, ATP-binding cassette transporter 1-4; ABCG2, ATP-binding cassette subfamily G member 2; IRF3, interferon regulatory factor 3; FR, folate receptor; RFC1, reduced folate carrier 1.

HMGB1 contributes to cardiometabolic diseases. HMGB1-mediated macrophage polarization toward a proinflammatory phenotype via TLR2 and TLR4 signaling promotes myocardial inflammation and cardiac remodeling.^209^ HMGB1 expression is increased in macrophages and smooth muscle cells within atherosclerotic lesions compared with normal human arteries.^210^ Excessive HMGB1 release from smooth muscle cells, macrophages, endothelial cells, foam cells, and activated platelets upregulates major proatherogenic factors, including intracellular adhesion molecule 1 (ICAM-1), vascular cell adhesion molecule 1 (VCAM-1), E-selectin, granulocyte colony-stimulating factor, TNF-*α*, monocyte chemoattractant protein-1 (MCP-1), IL-8, plasminogen activator inhibitor 1, and tissue plasminogen activator.^211^^,^^212^ Circulating HMGB1 is significantly higher in patients with obstructive sleep than in healthy subjects and increases further in those with overt atherosclerotic disease.^213^ In type 2 diabetes, circulating HMGB1 is independently associated with carotid plaque vulnerability.^214^ HMGB1 is also increased in obesity, likely due to excessive release from necrotic adipocytes and adipocytes exposed to proinflammatory stimuli such as TNF-*α*, which contribute to local and systemic inflammation in obesity and insulin resistance.^215^, ^216^, ^217^, ^218^ Animal models of septic hyperglycemia further support a close relationship between HMGB1 and glucose homeostasis.^219^

The first evidence that HMGB1 may be a methotrexate target came from screening for methotrexate-binding proteins using the T7 phage display system.^220^^,^^221^ This identified a region of HMGB1 as a possible direct binding site for methotrexate. The binding of methotrexate to HMGB1 through the AGER-binding region (K149-V175) interfered with HMGB1-AGER signaling and prevented HMGB1-mediated TNF-*α* release and mitogenic activity in RAW 264.7 mouse macrophages. Although methotrexate binding to regions involved in other receptors, such as TLRs or interleukin receptors, has no significant biological effects, these findings suggest that blocking HMGB1-AGER signaling may contribute to methotrexate pharmacology. The dissociation constant between methotrexate and HMGB1 at a site in the AGER-binding region (mean ± SE: 0.24 ± 0.01 *μ*mol/L) is similar to circulating methotrexate concentrations during low-dose treatment.^129^ HMGB1 mRNA expression in synovial tissue from patients with rheumatoid arthritis is also lower in those treated with methotrexate than in those receiving other disease-modifying antirheumatic drugs (DMARDs).^222^ Transfection of fibroblast-like synoviocytes with HMGB1 small interfering RNA prolongs cell doubling time and reduces migration, invasion, and expression of MMP-2 and MMP-13, which contribute to tissue breakdown, angiogenesis, inflammation, and oxidative stress in rheumatoid arthritis.^223^

Several studies highlight the beneficial effects of HMGB1 inhibitors in age-related cardiometabolic and neurodegenerative diseases (Fig. 7). Metformin inhibits vascular smooth muscle cell proliferation and migration by inducing miR-142-3p–mediated inhibition of HMGB1 expression.^224^ Glycyrrhizic acid, another HMGB1 inhibitor, significantly reduces serum lipid concentrations and atherosclerotic plaque burden in animal models.^225^ In rats with experimental diabetes, glycyrrhizin reduces renal injury and circulating glucose.^226^

Recent studies also support targeting HMGB1 in experimental models of Alzheimer and Parkinson disease. In a mouse model, apolipoprotein E4 (APOE4), a key driver of tau pathology and neurodegeneration in Alzheimer disease, induced nucleocytoplasmic translocation and extracellular release of HMGB1 from hippocampal neurons.^227^ This was associated with marked hippocampal gliosis, especially in older mice. HMGB1 inhibitors reduced APOE4-driven gliosis and neurodegeneration.^227^ In a zebrafish model of Parkinson disease, glycyrrhizic acid reduced apoptosis and locomotor impairment.^228^

### F Effects on gut microbiota

The gut microbiota, about 90% of which consists of *Bacteroidetes* and *Firmicutes*, plays a crucial role in maintaining intestinal barrier integrity, nutrient absorption, and vitamin and amino acid synthesis, and in regulating local and systemic inflammation and immune pathways.^229^, ^230^, ^231^ Human aging is associated with significant changes in the gut microbiota, including reduced diversity and loss of commensals such as *Bacteroides*, *Bifidobacterium*, and *Firmicutes*, along with increased abundances of *Proteobacteria* and other opportunistic microbes, including *Fusobacterium*, *Parabacteroides*, and *Ruminococcaceae*.^232^ This dysbiosis increases intestinal permeability, promotes systemic absorption of toxins, and favors a proinflammatory state associated with age-related cardiometabolic and neurodegenerative diseases (Fig. 8).^233^, ^234^, ^235^ Several drugs can alter microbial diversity and stability, potentially influencing disease risk.^236^^,^^237^

**Fig. 8:**
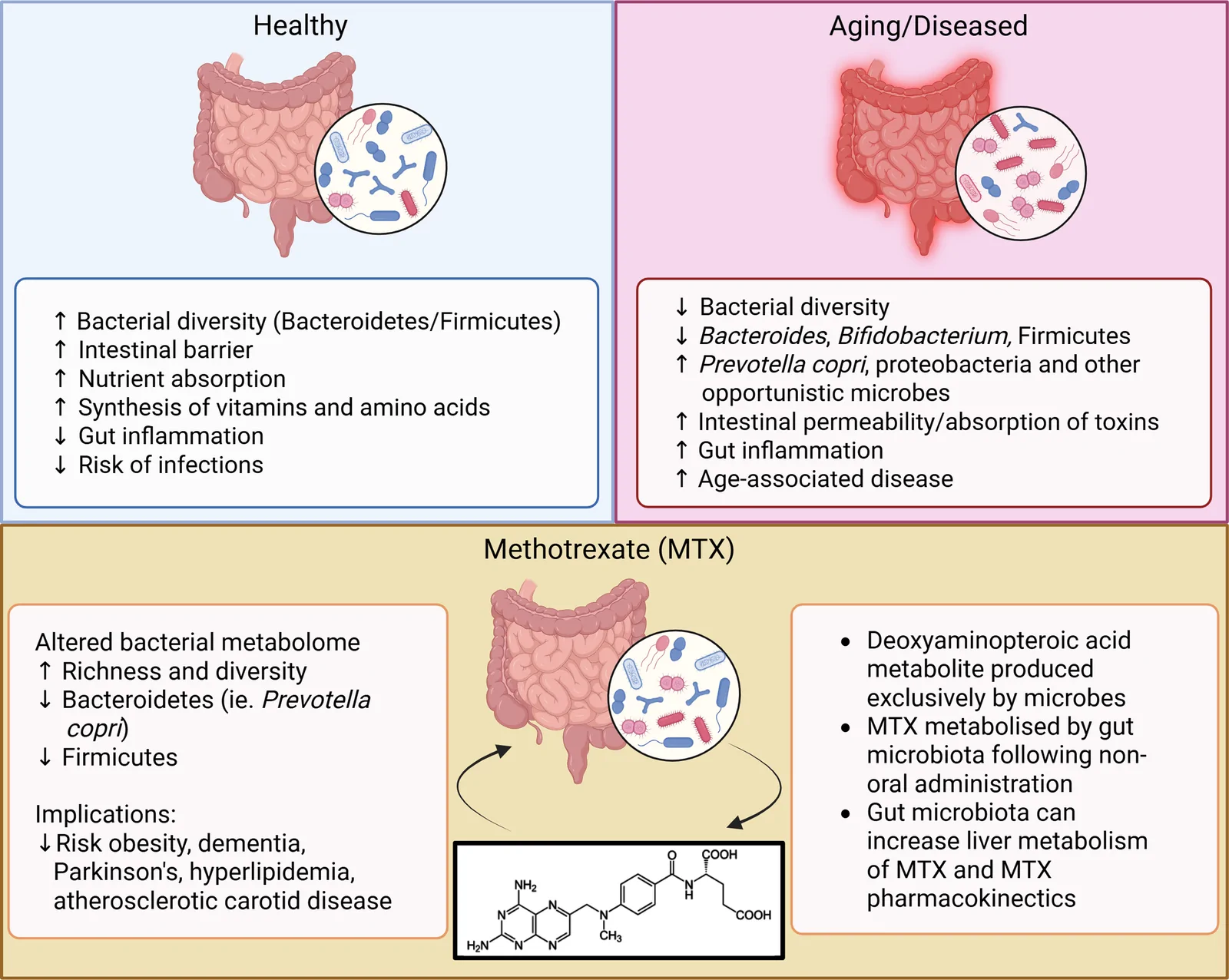
Interplay between methotrexate (MTX) and the gut microbiota and the potential effects on age-related cardiometabolic and neurodegenerative diseases. An aging/diseased gut has an altered bacterial microbiome, with decreased bacterial diversity and increased intestinal permeability and gut inflammation. MTX treatment alters the bacterial metabolome, resulting in increased bacterial richness and diversity and a decreased risk of obesity, dementia, and atherosclerotic carotid disease. In turn, MTX is metabolized by the gut microbiota following nonoral administration, which can increase hepatic metabolism of both MTX and its pharmacokinetics.

Methotrexate influences dysbiosis and the abundance of specific microbes. In germ-free mice colonized with stool from a healthy subject, methotrexate (1 mg/kg and 50 mg/kg daily for 4 days) reduced *Bacteroides* abundance and altered bacterial genera and amplicon sequence variants across *Actinobacteria*, *Firmicutes*, and *Proteobacteria*. Among bacterial isolates commonly found in the human gut microbiota, *Bacteroides* are more sensitive to methotrexate than *Firmicutes*, *Actinobacteria*, and *Proteobacteria*.^238^ Metabolomic studies show effects on purine and pyrimidine metabolism, likely via inhibition of bacterial DHFR, as well as on cysteine and methionine metabolism, arginine biosynthesis, and the citrate cycle. In patients with rheumatoid arthritis, methotrexate responders show significant reductions in the abundances of *Bacteroidetes* and *Prevotella copri*, a *Bacteroidetes* member associated with the disease.^239^ Colonization of germ-free mice with methotrexate-treated stool reduces host immune activation after a dextran sodium sulfate challenge.^238^ A significant reduction in *P copri* abundance has also been reported in 39% of patients with rheumatoid arthritis treated with methotrexate for 3 months at a mean weekly dose of 19 mg.^240^ These effects may be dose-dependent: low-dose methotrexate increases bacterial richness and diversity, with higher *Firmicutes* and lower *Bacteroidetes*, whereas high-dose treatment reduces bacterial diversity and favors higher *Bacteroidetes* and lower *Firmicutes*.^241^, ^242^, ^243^, ^244^

The effects of methotrexate on *P copri*, *Firmicutes*, and *Bacteroidetes* may have implications for age-related cardiometabolic and neurodegenerative diseases. Obesity has been associated with increased proliferation of *Bacteroidetes*, which promotes overproduction of lipopolysaccharides and short-chain fatty acids that activate TLR-4 and proinflammatory JAK/STAT and NF-*κ*B signaling.^245^ This mechanism may also explain the associations between *Bacteroidetes* and the risk of Alzheimer and Parkinson disease.^246^, ^247^, ^248^ A relative increase in *Bacteroidetes* is also associated with higher acetate production, which activates the parasympathetic nervous system, stimulates pancreatic *β* cells, and increases ghrelin secretion.^249^^,^^250^ Branched-chain amino acids are associated with the presence of *P copri* and *Bacteroides vulgatus* in the gut.^251^ In mice fed a high-fat diet, gavage with *P copri* increased serum branched-chain amino acid levels and promoted insulin resistance. In coronary artery disease, *Bacteroides* abundance is associated with total and LDL-cholesterol concentrations, and *P copri* with LDL-cholesterol levels.^252^ Patients with hyperlipidemia and atherosclerotic carotid disease also show significantly higher fecal *P copri* abundance and circulating trimethylamine N-oxide (TMAO) levels, a proatherosclerotic gut-derived factor, than those without carotid disease, and both correlate positively with carotid plaque scores.^253^ A 1-week treatment with the natural compound puerarin (150 mg/d orally) reduced *P copri* abundance and TMAO concentrations in these patients and in ApoE^-/-^ mice fed a high-fat diet.

A recent study in mice further highlights the complex interaction between methotrexate and the gut microbiota by describing new pharmacokinetic and pharmacodynamic effects.^254^ After an oral dose of 50 mg/kg, germ-free mice had significantly higher plasma methotrexate concentrations than specific pathogen-free mice, underscoring the microbiome’s critical role in gastrointestinal absorption. The methotrexate metabolite, deoxyaminopteroic acid, was detected only in plasma from specific pathogen-free mice, supporting the view that this metabolite is microbially produced and can enter the circulation. Similar plasma concentrations after intraperitoneal and oral methotrexate administration suggest ongoing microbial metabolism following nonoral administration via enterohepatic circulation. The area under the curve for 7-hydroxy-methotrexate, produced by hepatic aldehyde oxidase, was relatively higher than that of methotrexate in specific pathogen-free mice, suggesting that the gut microbiota can increase hepatic methotrexate metabolism. When germ-free mice were colonized with fecal communities from 2 treatment-naïve rheumatoid patients and then given oral administration at the same dose (50 mg/kg), significant differences in colonization state were observed at peak plasma concentrations of the parent drug, but not at peak concentrations of 7-hydroxy-methotrexate or deoxyaminopteroic acid.^254^ These findings indicate a substantial effect of the gut microbiota on methotrexate pharmacokinetics, though not through first-pass metabolism. Incubation of unlabeled and d_3_-labeled methotrexate with 2 human gut bacterial isolates that metabolize methotrexate, *Clostridium asparagiforme and Clostridium symbiosum*, and 1 that does not, *Bacteroides thetaiotaomicron*, also identified a new metabolite, p-methylaminobenzoyl-L-glutamic acid, in blood. Another metabolite, 6-methylpteridine-2,4-diamine, was detected in vitro but not in vivo.

### G Overall assessment of the available evidence

Across age-related cardiometabolic and neurodegenerative phenotypes, the adenosine axis provides the strongest mechanistic support for methotrexate. A_2A_/A_3_ receptor engagement is required for anti-inflammatory effects in vivo and is consistent with adenosine-mediated suppression of leukocyte activation and cytokine release. Adenosine signaling also mediates atheroprotective effects, including enhanced cholesterol efflux, reduced foam cell transformation, attenuated endothelial activation, vasodilation, enhanced insulin sensitivity, and neuroprotection. Preclinical evidence for AMPK-dependent vascular protection is moderate to strong, with methotrexate limiting disturbed-flow endothelial inflammation and plaque formation by regulating the AMPK-yes-associated protein/transcriptional coactivator with PDZ-binding motif and by inducing AMPK-regulated cytoprotective genes. Methotrexate-mediated AMPK activation also improves insulin sensitivity, whereas neuroprotective effects have mainly been observed with other AMPK activators. By contrast, the AMPK-independent effects of AICAR on the LDL receptor, oxidized LDL uptake, and the endoplasmic reticulum stress response remain largely cellular and hypothesis-generating, although LRRK2 downregulation provides more direct evidence relevant to Parkinson disease models. JAK/STAT and HMGB1-AGER inhibition are compelling but remain predominantly mechanistic and require dedicated in vitro and in vivo studies with methotrexate. Microbiome-mediated effects are increasingly supported by humanized mouse models and rheumatoid arthritis cohorts, but their relevance to age-related cardiometabolic and neurodegenerative diseases remains inferential.

## V Low-dose methotrexate and age-related cardiometabolic and neurodegenerative diseases

The following sections discuss, for each key age-related cardiometabolic and neurodegenerative disease, the main pathophysiological mechanisms, the potential relevance of each methotrexate target recently identified in experimental studies and described in Section IV, and the results of human studies examining associations between low-dose methotrexate and each condition.

### A Age-related cardiometabolic diseases and risk factors

#### 1 Atherosclerosis

##### a Pathophysiology

Atherosclerosis is a chronic disease of the arterial wall, characterized by maladaptive fibro-inflammatory remodeling and initiated by the binding of apolipoprotein B-containing lipoproteins to extracellular matrix proteoglycans in the intima.^255^ Endothelial dysfunction, characterized by structural and functional alterations in endothelial cells and reduced NO synthesis, increases permeability, adhesion molecule expression, and chemokine gradients, thereby promoting the recruitment of monocytes and T cells.^256^ In the intima, macrophages and phenotypically switched vascular smooth muscle cells internalize modified lipoproteins, thereby becoming foam cells, a process that reflects not only increased lipid uptake but also impaired cholesterol efflux.^257^ This abnormal cell phenotype promotes excessive cytokine production, oxidative stress, and innate immune activation, thereby contributing to the development of atherosclerotic plaques.^258^ Adaptive immunity plays a key role in shaping plaque biology. T-helper 1-derived IFN-gamma promotes macrophage activation and impairs fibrous-cap repair. Systemic drivers such as dysregulated myelopoiesis and clonal hematopoiesis can further amplify plaque inflammation.^259^ Atherosclerotic plaque progression involves cell death and defective efferocytosis, resulting in lipid-rich necrotic cores, cholesterol crystals, calcification, and, in advanced lesions, intraplaque hemorrhage.^260^ Matrix remodeling and the smooth muscle-derived extracellular matrix form a fibrous cap. Inflammatory mediators and proteases thin this cap, predisposing it to rupture, superimposed thrombosis, and the onset of acute cardiovascular events, which are the clinical manifestations of atherosclerosis.^255^

##### b Potential therapeutic effects of methotrexate targeting pathophysiological mechanisms

Several recently identified methotrexate targets are directly relevant to the pathophysiology of atherosclerosis. Methotrexate-mediated adenosine accumulation promotes reverse cholesterol transport from macrophages via A_2A_ receptor-dependent upregulation of cholesterol 27-hydroxylase, ABCA1, and ABCG1, thereby reducing foam cell transformation by 30%–40% (Fig. 3; Table 1).^94^, ^95^, ^96^^,^^261^ Impaired macrophage cholesterol efflux capacity independently predicts incident atherosclerotic cardiovascular events beyond conventional lipid parameters, ie, high-density lipoprotein (HDL)-cholesterol.^262^ Therefore, pharmacological strategies that restore these efflux pathways may translate into meaningful atheroprotective effects, particularly in patients with persistent residual cardiovascular risk despite treatment with conventional lipid-lowering therapies.^263^ Methotrexate also increases the expression of the anti-inflammatory microRNA-181b through A_3_ receptor-mediated regulation of the *MIR181B-2* promoter by mothers against decapentaplegic homologs 3 and 4, an effect observed at clinically relevant concentrations (Table 1).^97^, ^98^, ^99^^,^^129^ The pathophysiological relevance of targeting microRNA-181b is supported by its inhibitory effects on importin-*α*3, thereby restricting NF-*κ*B nuclear translocation in endothelial cells and reducing VCAM-1, E-selectin, and leukocyte adhesion. Furthermore, systemic delivery of microRNA-181b inhibits arterial wall inflammation and atherosclerotic progression in *Apoe*^*-/-*^ mice.^97^^,^^264^^,^^265^ Although not tested directly, this pathway may at least partly explain the atheroprotective effects of methotrexate in animal models of in-stent atherosclerosis, mediated by reductions in neointimal area and thickness, increases in fibrous cap thickness, and reductions in circulating IL-6 and ICAM-1.^100^ These effects are consistent with reductions in plaque burden, arterial wall inflammation, and lesion stability.^255^

AMPK activation following methotrexate treatment (1 mg/kg/wk for 12–16 weeks) reduces vasculopathy and the number of animals that develop myocardial infarction in experimental models of atherosclerosis.^144^ Furthermore, it upregulates atheroprotective genes, including manganese superoxide dismutase and heme oxygenase-1, reduces inflammation in perivascular adipose tissue, improves endothelium-dependent vasodilation, and suppresses yes-associated protein and transcriptional coactivator with PDZ-binding motif-mediated plaque formation under conditions of disturbed flow, all at concentrations comparable to those measured during low-dose treatment (Fig. 4).^144^, ^145^, ^146^, ^147^ The atheroprotective effect of AMPK activation is further supported by translational evidence. Endothelial AMPK activity declines with aging, insulin resistance, and disturbed blood flow. This decline is associated with impaired eNOS phosphorylation and activity, reduced autophagy, and transition toward a proinflammatory endothelial cell phenotype.^266^^,^^267^ By lowering the threshold for AMPK activation in the arterial wall, methotrexate may counteract several key pathophysiological steps that precede overt atherosclerosis. AMPK activation also increases macrophage autophagy and reduces plaque vulnerability and instability through the AGER/liver kinase B1/AMPK1/sirtuin 1 pathways.^268^^,^^269^ Furthermore, AMPK is a critical regulator of sterol and lipid metabolism, with experimental evidence showing that its activation inhibits HMG-CoA reductase and cholesterol synthesis through direct phosphorylation of the conserved C-terminal serine residue (Ser872 in the human enzyme).^148^ Disruption of AMPK-dependent HMG-CoA inhibition in mouse hematopoietic stem and progenitor cells increases cholesterol and triglyceride synthesis during high-carbohydrate feeding, leading to cholesterol dysregulation, myelopoiesis, and accelerated atherosclerosis.^270^ AMPK activation also suppresses lipogenesis and enhances lipid disposal, including inhibition of sterol regulatory element-binding pathways, increased fatty acid oxidation via acetyl-CoA carboxylase phosphorylation, and increased expression of hepatobiliary cholesterol transporters such as ATP-binding cassette subfamily G members 5 and 8.^149^

AICAR, accumulated through ATIC inhibition, exerts atheroprotective effects by increasing LDL receptor mRNA expression and protein levels (Fig. 5).^167^ The increased mRNA levels, partly due to stabilization, result from the interaction between AICAR and the 3'-untranslated region of LDL receptor mRNA and depend on ERK1/2 activation but not on AMPK activation. AICAR also suppresses PPAR*γ* expression and PPAR*γ* target gene activation during monocyte-to-macrophage differentiation, a process critical for oxidized LDL uptake and the heightened inflammatory response to oxidized LDL and saturated fatty acids.^168^ These effects are not influenced by AMPK inhibitors or *AMPK* silencing.

Pharmacological JAK/STAT inhibition with ruxolitinib and tofacitinib (Fig. 6) reduces the expression of the scavenger receptors CD36 and CD204, which mediate the uptake of modified LDL by lipopolysaccharide-activated murine macrophages, a critical step in atherogenesis.^184^ Tofacitinib also decreases lipid accumulation in THP-1–derived macrophages exposed to high lipid concentrations and IFN-gamma by increasing ABCA1 expression, which facilitates the transfer of intracellular free cholesterol and phospholipids to apolipoprotein A-I for new HDL particle formation.^185^^,^^186^ Therefore, JAK/STAT provides an additional mechanistic framework linking methotrexate to reduced foam cell formation (Fig. 6). Atheroprotective effects have also been reported in animal studies. A 9-week treatment with the JAK2 inhibitor fedratinib significantly reduced atherosclerotic lesions in the aortas of *Apoe*^*-/-*^ mice fed a Western diet.^189^ Similar effects were observed with ruxolitinib in rabbits subjected to aortic balloon injury and fed an atherogenic diet.^190^

The direct binding of methotrexate to HMGB1 through the AGER-binding region, with a dissociation constant comparable to circulating methotrexate concentrations during low-dose treatment, may attenuate plaque burden and prevent the release of multiple proatherogenic factors, including ICAM-1, VCAM-1, TNF-*α*, and MCP-1 (Fig. 7).^210^, ^211^, ^212^, ^213^, ^214^^,^^220^^,^^221^ The antidiabetic drug metformin inhibits, in a dose-dependent manner, vascular smooth muscle cell proliferation and migration, critical steps in atherogenesis,^271^ by inducing miR-142-3p–mediated inhibition of HMGB1 expression through the HMGB1 autophagy-related pathway.^224^ Glycyrrhizic acid, another HMGB1 inhibitor, reduces serum lipid concentrations and atherosclerotic plaque burden in Apoe^-/-^ mice fed a high-fat diet.^225^ These effects are associated with lower IL-17A and IL-6 and higher IL-10 (Fig. 7). The relevance of HMGB1 inhibition extends beyond the direct suppression of proatherogenic factors, as circulating HMGB1 concentrations independently predict major adverse cardiovascular events and correlate with histological markers of plaque vulnerability, such as fibrous caps and enlarged necrotic scores.^210^^,^^272^ Therefore, methotrexate-induced HMGB1 inhibition provides a mechanistic rationale for targeting a pathway of established prognostic significance in atherosclerosis.

Lastly, the methotrexate-induced reduction in *P copri* may attenuate atherogenesis by decreasing the gut microbiota’s production of the proatherosclerotic factor TMAO (Fig. 8).^239^^,^^252^^,^^253^ This observation aligns with the recognition of TMAO as an independent determinant of atherosclerotic disease risk in large prospective cohorts.^273^^,^^274^

Therefore, methotrexate may exert significant atheroprotective effects by attenuating endothelial activation, foam cell formation, vascular inflammation, plaque destabilization, and the gut microbiota’s production of proatherosclerotic factors.

##### c Human studies

Several observational studies have examined the association between low-dose methotrexate and the risk of atherosclerosis-related cardiovascular events, such as myocardial infarction and ischemic stroke, in patients with autoimmune diseases, especially rheumatoid arthritis. One of the early studies included 1240 adult patients with rheumatoid arthritis who were treated with methotrexate or other DMARDs.^275^ After an average follow-up of 6 years, methotrexate users (mean dose 13 mg weekly) had a significantly lower risk of cardiovascular mortality than those of other DMARDs (hazard ratio [HR], 0.3; 95% CI, 0.2–0.7).^275^ The effects were similar when analyses focused only on methotrexate users not receiving concomitant folic acid (HR, 0.5; 95% CI, 0.3–0.9) or statins (HR, 0.4; 95% CI, 0.3–0.8). In another study involving patients with psoriasis (*n* = 7615) and rheumatoid arthritis (*n* = 6707), methotrexate users had a significantly lower risk of atherosclerotic vascular disease compared with nonusers after adjusting for age, sex, diabetes mellitus, hypertension, dyslipidemia, and use of folic acid, vitamin B_6_, and vitamin B_12_ (psoriasis group relative risk [RR], 0.73; 95% CI, 0.55–0.98; rheumatoid arthritis group RR, 0.83; 95% CI, 0.71–0.96).^276^ In a systematic review and meta-analysis of 7 longitudinal observational cohort studies, 6 retrospective cohort studies, and 2 prospective cohort studies involving patients with rheumatoid arthritis, the use of methotrexate was associated with a significant reduction in the risk of cardiovascular disease (HR, 0.72; 95% CI, 0.53–0.97; I^2^ = 87.9%).^277^ The observed effect size for cardiovascular risk reduction with methotrexate in this meta-analysis was similar to that of well-established atheroprotective treatments, such as angiotensin-converting enzyme inhibitors (RR, 0.80; 95% CI, 0.70–0.91)^278^ and statins (OR, 0.69; 95% CI, 0.64–0.75),^279^ in intervention trials in patients without autoimmune diseases.

The results of these investigations prompted a randomized controlled trial, the Cardiovascular Inflammation Reduction Trial (CIRT), which assessed the effects of low-dose methotrexate versus placebo on cardiovascular outcomes.^280^ The study included 4786 patients with a high overall cardiovascular risk who did not have autoimmune disease. After a median follow-up of 2.3 years, there were no significant differences between groups in the composite endpoint of nonfatal myocardial infarction, nonfatal stroke, cardiovascular death, and hospitalization for unstable angina requiring urgent revascularization (HR, 0.96; 95% CI, 0.79–1.16).

#### 2 Heart failure

##### a Pathophysiology

Heart failure, a clinical syndrome often resulting from longstanding atherosclerotic and cardiometabolic risk factors,^5^^,^^281^ arises when myocardial injury or chronic hemodynamic stress activates maladaptive neurohormonal, inflammatory, metabolic, and structural responses culminating in cardiac pump dysfunction.^282^ Reduced forward output and/or elevated filling pressures activate the sympathetic and renin-angiotensin-aldosterone systems. This, in turn, increases heart rate, vasoconstriction, sodium retention, and cardiac afterload. Long-term activation of these systems also promotes left ventricular hypertrophy, myocyte apoptosis, arrhythmogenic remodeling, and interstitial fibrosis.^5^ In heart failure with reduced ejection fraction, loss of contractile units and progressive impairment of excitation-contraction coupling amplify pump failure over time.^283^ By contrast, in heart failure with preserved ejection fraction, systemic comorbidities, such as hypertension and diabetes mellitus, promote coronary microvascular inflammation and endothelial dysfunction, resulting in impaired NO signaling, increased cardiomyocyte stiffness, and concentric remodeling.^284^ Inflammatory cytokines, oxidative stress, mitochondrial energy deficits, and defective calcium handling further contribute to adverse remodeling, reduced cardiac reserve, and congestion, culminating in multiorgan dysfunction and episodic decompensation. Counter-regulatory natriuretic peptide pathways are often inadequate, and venous congestion with renal hypoperfusion perpetuates cardiorenal activation, further elevating filling pressures and increasing the risk of recurrent hospitalizations.^282^

##### b Potential therapeutic effects of methotrexate targeting pathophysiological mechanisms

Several mechanisms identified in Section IV may underpin the potential therapeutic effects of methotrexate in heart failure. Because endothelial dysfunction, vascular inflammation, oxidative stress, and neurohormonal activation contribute to ventricular remodeling and impaired cardiac reserve in heart failure, methotrexate-mediated adenosine signaling may support coronary and peripheral blood flow through A_2A_-dependent vasodilation by opening voltage-gated and ATP-sensitive potassium channels in vascular smooth muscle cells, thereby reducing peripheral vascular resistance and cardiac afterload (Fig. 3; Table 1).^101^ Additionally, A_2A_ receptor-mediated stimulation of eNOS increases NO synthesis. This is particularly relevant to heart failure with preserved ejection fraction, in which impaired NO signaling contributes to cardiomyocyte stiffness and concentric remodeling.^102^^,^^285^ Adenosine-mediated improvement in vascular homeostasis may enhance coronary microvascular function and tissue perfusion, processes that are often impaired in patients with cardiometabolic comorbidities.^286^ Adenosine also exerts direct cardioprotective effects that extend beyond its hemodynamic actions. A_2A_ receptor activation attenuates ischemia-reperfusion injury in experimental models by reducing neutrophil infiltration and infarct size and preserving postischemic left ventricular function.^287^^,^^288^ These effects are mediated, at least in part, by inhibition of the NOD-like receptor protein 3 inflammasome and suppression of pyroptosis in cardiomyocytes, pathways that are increasingly recognized as driving adverse cardiac remodeling in both ischemic and nonischemic heart failure.^288^^,^^289^ A_2A_ receptor signaling also promotes M2 macrophage polarization in myocardial tissues, shifting the inflammatory milieu toward a reparative phenotype, thereby favoring the resolution of inflammation and scar maturation over ongoing tissue necrosis.^288^^,^^290^

AMPK activation is also relevant because it induces cytoprotective genes, attenuates vascular and perivascular inflammation, and improves substrate utilization and glucose handling, mechanisms that could alleviate energetic inefficiency and inflammatory remodeling in the failing myocardium (Fig. 4).^104^^,^^105^^,^^144^, ^145^, ^146^, ^147^^,^^151^^,^^291^^,^^292^ The relevance of AMPK activation in heart failure extends to the regulation of cardiac autophagy and mitophagy. In the failing myocardium, impaired clearance of damaged mitochondria further contributes to energy failure, oxidative stress, and cardiomyocyte death.^293^ AMPK-mediated phosphorylation of Unc-51-like kinase 1 initiates autophagosome formation, whereas concomitant inhibition of mTORC1 removes the constitutive block on autophagic flux, thereby restoring mitochondrial quality control.^294^^,^^295^ AMPK activation also attenuates pathological cardiac hypertrophy and fibrosis in experimental models of pressure-overload–induced heart failure by suppressing protein synthesis via the mTOR pathway and reducing reactive oxygen species production.^296^^,^^297^ Since methotrexate lowers the threshold for AICAR-induced AMPK activation, similar cardioprotective effects may occur via the methotrexate-ATIC-AICAR axis.^104^

JAK/STAT inhibition may counteract excess endothelin-1 production, a potent vasoconstrictor implicated in the progression of heart failure (Fig. 6).^189^^,^^298^ Since cytokine-driven JAK/STAT signaling also contributes more broadly to inflammatory and fibrotic responses, its inhibition provides an additional mechanism by which methotrexate might attenuate maladaptive cardiac remodeling.^299^ In this context, IL-6–mediated STAT3 activation in cardiac fibroblasts promotes myocardial fibrosis through dysregulated collagen synthesis and extracellular matrix expansion, leading to increased ventricular stiffness, particularly in heart failure with preserved ejection fraction.^300^^,^^301^ Pharmacological JAK2 inhibition attenuates angiotensin II-induced cardiac hypertrophy and fibrosis in experimental models, with concomitant reductions in TGF-*β*1 signaling and improvements in diastolic function.^302^^,^^303^ The JAK/STAT inhibitor ruxolitinib also suppresses the senescence-associated secretory phenotype in aged cardiomyocytes, potentially limiting the age-dependent inflammatory burden that accelerates progression from compensated hypertrophy to overt heart failure.^304^

The inhibition of HMGB1, which promotes proinflammatory macrophage activation and cardiac remodeling through TLR2 and TLR4 signaling, represents another potentially relevant mechanism (Fig. 7).^209^ Circulating HMGB1 concentrations are significantly elevated in patients with both acute and chronic heart failure and correlate with left ventricular dysfunction, natriuretic peptide concentrations, and disease severity.^305^^,^^306^ HMGB1 also promotes cardiomyocyte apoptosis and activates cardiac fibroblasts, contributing to interstitial fibrosis and progressive cardiac chamber dilatation.^307^^,^^308^ The ability of methotrexate to bind directly to HMGB1 through the AGER-binding region at clinically relevant concentrations therefore provides a rationale for interrupting this pathway at a proximal point, potentially limiting both the inflammatory and fibrotic components of cardiac remodeling.^129^^,^^220^

Finally, gut microbiota dysbiosis, a recently recognized feature of heart failure, contributes to systemic inflammation through increased intestinal permeability, bacterial translocation, and elevated circulating levels of lipopolysaccharides and TMAO.^309^^,^^310^ Elevated TMAO concentrations independently predict mortality and hospitalization in patients with heart failure, and dietary TMAO supplementation worsens cardiac remodeling and ventricular dysfunction in experimental models.^311^ The methotrexate-induced reduction in *P copri*, a major TMAO-producing species, may therefore attenuate the detrimental effects of this gut-heart signaling axis.^239^

Taken together, the mechanistic evidence supports a biologically plausible protective role for methotrexate in heart failure by modulating coronary microvascular inflammation, endothelial NO deficiency, inflammatory-fibrotic remodeling, and metabolic inefficiency, and by attenuating specific gut-heart pathways.

##### c Human studies

A retrospective cohort study examined 9889 patients with an International Classification of Diseases, Ninth Revision code for rheumatoid disease or other autoimmune diseases, along with 9889 controls, first matched for sex and race, then by selecting the individual closest in age.^312^ Over a median follow-up of 8.7 years, the incidence of heart failure was 21% higher in patients with rheumatoid arthritis (95% CI, 3–42; *P* = .02). The use of antirheumatic drugs showed variable associations with incident heart failure. Specifically, the use of methotrexate was significantly associated with a lower risk of incident heart failure (adjusted OR [aOR], 0.75; 95% CI, 0.59–0.96). By contrast, there was no significant influence of treatment with other csDMARDs, TNF-*α* inhibitors, corticosteroids, antimalarials, or biologic/small-molecule DMARDs. In another observational study of 795 patients with rheumatoid arthritis followed for an average of 9.7 years, current use of methotrexate, but not other csDMARDs, biologics, or corticosteroids, was associated with a significant decrease in incident heart failure (HR, 0.5; 95% CI, 0.3–0.9).^313^

Intervention trials of methotrexate in patients with heart failure but without autoimmune diseases have also been conducted. In a randomized, placebo-controlled study involving 71 patients with heart failure, a 12-week treatment with 7.5 mg of methotrexate once weekly was associated with significant improvements in 6-minute walk test distance and quality-of-life scores (both *P* < .05).^314^ These effects were not associated with changes in inflammatory mediators, except for a significant reduction in the proinflammatory MCP-1 (*P* = .037) and an increase in the anti-inflammatory soluble IL-1 receptor antagonist (*P* = .004). In another randomized, placebo-controlled study of 50 patients with ischemic heart failure, a 12-week methotrexate treatment (7.5 mg/wk) did not significantly affect the 6-minute walk test distance or the New York Heart Association class.^315^ In this study, both the methotrexate and placebo arms received folic acid treatment (5 mg/wk).^315^

#### 3 Diabetes mellitus

##### a Pathophysiology

Diabetes mellitus comprises heterogeneous disorders unified by chronic hyperglycemia resulting from absolute or relative insulin deficiency and impaired insulin action. Type 1 diabetes is characterized by immune-mediated *β*-cell loss. Current evidence supports a bidirectional process in which *β*-cell intrinsic stress (eg, endoplasmic reticulum stress, inflammatory signaling, and altered antigen presentation) compromises *β*-cell integrity and can amplify autoimmunity.^316^ Type 2 diabetes develops when obesity-associated insulin resistance in skeletal muscle and liver, resulting from ectopic lipid deposition, mitochondrial dysfunction, and low-grade systemic inflammation, is no longer compensated by adaptive *β*-cell hypersecretion.^317^ Progressive *β*-cell failure results from glucotoxicity, lipotoxicity, oxidative stress, impaired incretin responses, and apoptosis, leading to increased fasting and postprandial serum glucose concentrations.^318^ Genetic susceptibility and adipose dysfunction have been shown to play important roles in modulating disease progression and phenotypic heterogeneity.^319^

##### b Potential therapeutic effects of methotrexate targeting pathophysiological mechanisms

Multiple methotrexate targets discussed in Section IV are relevant to the pathophysiology of diabetes mellitus. Adenosine receptor activation enhances glucose homeostasis through several mechanisms: (1) partial A_1_ receptor agonists reduce circulating free fatty acids, triglycerides, and insulin, and (2) A_1_ receptor agonists increase insulin-stimulated glucose uptake in skeletal muscle in a dose-dependent manner (Fig. 3; Table 1).^106^^,^^107^ A_2A_ receptor activation improves glucose regulation, decreases inflammatory markers in adipose tissue, enhances insulin sensitivity in high-fat diet models, and protects *β* cells from cytokine-induced apoptosis and promotes their regeneration in experimental models of diabetes, thereby preserving endogenous insulin secretion.^103^^,^^108^^,^^112^^,^^320^^,^^321^ A_2A_ receptor activation also protects against diabetic neuropathy,^109^ retinopathy,^110^ and nephropathy.^111^ The A_2B_ receptor, highly expressed in skeletal muscle and brown adipose tissue, is particularly relevant because its activation increases brown fat metabolic activity, reduces oxidative stress and tissue senescence, and increases energy expenditure and basal and insulin-stimulated glucose uptake in human myocytes (Fig. 3; Table 1).^113^ This suggests a possible influence of methotrexate on the interplay among aging, adipose dysfunction, and impaired peripheral glucose disposal.^322^ In human biopsies, A_2B_ receptor expression is lower in overweight than in lean individuals, negatively correlated with age, and inversely related to aging-associated pathways in skeletal muscle.^113^ Notably, A_2B_ receptor activation not only enhances glucose uptake but also opposes sarcopenia, a frequently overlooked contributor to worsening insulin resistance in older adults with type 2 diabetes.^113^^,^^322^^,^^323^

Methotrexate-mediated AMPK activation stimulates glucose uptake via GLUT4 upregulation and enhances lipid utilization in skeletal muscle (Fig. 4), as evidenced by palmitate oxidation, with effects confirmed in diabetic mice receiving a 4-week low-dose methotrexate treatment (0.5 mg/kg weekly).^104^^,^^105^ In addition to upregulating GLUT4, AMPK phosphorylates acetyl-CoA carboxylase, thereby reducing malonyl-CoA concentrations, relieving the inhibition of carnitine palmitoyltransferase 1, and enhancing mitochondrial fatty acid oxidation in skeletal muscle and the liver.^134^ This metabolic switch improves ectopic lipid disposal, a key driver of hepatic and muscular insulin resistance.^324^ AMPK activation also suppresses hepatic gluconeogenesis through phosphorylation of the cAMP response element-binding protein-regulated transcription coactivator 2 and downregulation of phosphoenolpyruvate carboxykinase and glucose-6-phosphatase, mechanisms that are well characterized for metformin and that may at least partly explain the observed effects of methotrexate on fasting glucose in diabetic models.^105^^,^^325^ The translational relevance of AMPK activation is reinforced by Mendelian randomization studies linking genetic variants that increase AMPK activity to lower fasting glucose, reduced risk of type 2 diabetes, and improved cardiometabolic outcomes.^326^

In human macrophages, AICAR exerts AMPK-independent protective effects by attenuating the endoplasmic reticulum stress response following saturated fatty acid treatment (Fig. 5), a critical step in the development of insulin resistance and type 2 diabetes.^169^^,^^327^ Furthermore, *β*-cell dysfunction in both islet lipotoxicity and glucotoxicity is driven by unresolved unfolded protein response and activation of the integrated stress response via signaling by pancreatic endoplasmic reticulum kinase, activating transcription factor 6, and inositol-requiring enzyme 1*α*.^328^^,^^329^ By attenuating these pathways independently of AMPK, AICAR accumulation during methotrexate therapy may help preserve *β*-cell function in the context of chronic nutrient excess.^169^

Combined JAK/STAT inhibition with tofacitinib and aspirin (an NF-*κ*B inhibitor) reduces proinflammatory cytokines, insulin resistance, and serum glucose concentrations while increasing insulin secretion and GLUT4 expression.^191^ In patients with rheumatoid arthritis and type 2 diabetes, 6 months of tofacitinib reduced insulin resistance in an open-label proof-of-concept study.^192^ In retrospective studies, baricitinib, but not tofacitinib, reduced glycated hemoglobin after 6 months in patients with rheumatoid arthritis and type 2 diabetes.^193^ In other studies, 24-week tofacitinib treatment reduced insulin resistance in patients with rheumatoid arthritis without overt type 2 diabetes but with high baseline insulin resistance.^194^ JAK/STAT inhibition has also shown beneficial effects in diabetic nephropathy, a leading cause of renal replacement treatment and kidney transplant worldwide.^330^ In a phase 2, double-blind, randomized controlled trial in patients with type 2 diabetes, 24-week treatment with baricitinib reduced the urine albumin-to-creatinine ratio by 41% compared with placebo (Fig. 6).^195^ In this context, JAK/STAT inhibition is also emerging as a promising approach for diabetes-related complications, as sustained activation of this pathway, particularly through IL-6-JAK2-STAT3 signaling, promotes adipose tissue inflammation, suppresses insulin receptor substrate 1, and impairs insulin signaling in skeletal muscle and liver.^331^ STAT1 activation in pancreatic *β* cells, driven by type I and type II IFNs, contributes to *β*-cell death in both type 1 and type 2 diabetes, and JAK inhibition has been shown to preserve *β*-cell mass in experimental models.^332^ The reported improvement in glucose homeostasis with baricitinib and tofacitinib supports the pathophysiological relevance of this axis.^191^, ^192^, ^193^, ^194^, ^195^

HMGB1 inhibition by glycyrrhizic acid reduces glucose and proinflammatory cytokine concentrations (TNF-*α*, IL-6, and IL-1*β*) and attenuates renal injury in rats with streptozotocin-induced diabetes mellitus (Fig. 7).^226^ The pathophysiological role of HMGB1 in diabetes is further supported by studies reporting that circulating HMGB1 concentrations are elevated in patients with type 2 diabetes and are independently associated with insulin resistance, *β*-cell dysfunction, and microvascular complications, including diabetic nephropathy and retinopathy.^333^

The methotrexate-mediated reduction of *P copri* may further improve insulin sensitivity, given the established role of this microbe in increasing circulating branched-chain amino acid levels and promoting insulin resistance (Fig. 8).^251^ This role is further supported by studies reporting that transplantation of *P copri* increases serum concentrations of glucose, insulin, and IL-1*β* in high-fat diet-induced obese mice but not in normal mice.^334^

Together, these data support the proposition that methotrexate is a potential modulator of insulin resistance, adipose inflammation, disordered lipid handling, glucotoxicity-associated stress pathways, diabetic end-organ injury, and changes in gut microbiota composition.

##### c Human studies

One of the first observational studies investigating the association between methotrexate and incident diabetes included 13,905 patients with rheumatoid arthritis or psoriasis who were commenced on different DMARDs.^335^ After a mean follow-up of 5.8 months, methotrexate use was not associated with a significant reduction in incident diabetes after adjustment for the type of autoimmune disease, age, sex, comorbidities, and other medications, including prior use of hydroxychloroquine, methotrexate, other nonbiologic immunosuppressive drugs, and glucocorticoids (HR, 0.77; 95% CI, 0.53–1.13). Subsequent studies have reported a protective effect of methotrexate against both type 1 and type 2 diabetes. In a systematic review and meta-analysis of 16 studies of patients with rheumatoid arthritis, methotrexate use was associated with a significant reduction in the risk of type 2 diabetes (RR, 0.13; 95% CI, 0.08–0.22; I^2^ = 98%).^336^ A similar negative association between methotrexate and both type 1 and type 2 diabetes was reported in another systematic review and meta-analysis of 15 studies (7 on methotrexate) involving 552,019 patients with rheumatoid arthritis (HR, 0.81; 95% CI, 0.75–0.87; I^2^ = 7.6%).^337^ In a recent population study of 69,799 patients with rheumatoid arthritis, methotrexate use was associated with a significant reduction in the incidence of type 1 and type 2 diabetes (aOR, 0.84; 95% CI, 0.78–0.92).^338^ A systematic review and meta-analysis of the oral antidiabetic drug metformin reported a 50% reduced risk (95% CI, 0.38–0.65) of developing type 2 diabetes in patient populations mainly without autoimmune diseases.^339^

#### 4 Dyslipidemia

##### a Pathophysiology

Dyslipidemia is characterized by an imbalance among hepatic lipoprotein production, intravascular remodeling, and receptor-mediated clearance. Such an imbalance leads to increased circulating concentrations of atherogenic apolipoprotein B-containing particles, specifically very-LDL (VLDL), LDL, and lipoprotein(a). Impaired flux through the LDL receptor pathway, with consequent increases in circulating LDL particle residence time and retention in the arterial wall, is a key pathophysiological mechanism of dyslipidemia.^340^ In insulin-resistant states, “selective hepatic insulin resistance” promotes VLDL apolipoprotein-B hypersecretion, while reduced lipoprotein lipase activity and inhibition by apolipoprotein C-III and angiopoietin-like 3 impair triglyceride-rich lipoprotein lipolysis, thereby enriching remnant cholesterol and small, dense LDL.^341^ Low concentrations of atheroprotective HDL-cholesterol, another common feature of dyslipidemia, often reflect accelerated catabolism of triglyceride-enriched HDL and qualitative dysfunction of HDL, thereby reducing reverse cholesterol transport and anti-inflammatory capacity.^342^

##### b Potential therapeutic effects of methotrexate targeting pathophysiological mechanisms

The mechanisms by which methotrexate may exert beneficial effects on lipid metabolism involve several recently identified targets. As previously described, adenosine-mediated A_2A_ receptor activation upregulates cholesterol efflux pathways, including cholesterol 27-hydroxylase, ABCA1, and ABCG1, thereby promoting reverse cholesterol transport (Fig. 3; Table 1).^94^, ^95^, ^96^ This effect is particularly relevant to dyslipidemic states characterized not only by increased concentrations of atherogenic lipoproteins but also by impaired cellular cholesterol disposal and dysfunctional HDL biology.^343^

As previously discussed, AMPK activation inhibits HMG-CoA reductase and cholesterol synthesis.^148^ Disruption of AMPK-dependent HMG-CoA inhibition increases cholesterol and triglyceride synthesis, promotes cholesterol dysregulation, and accelerates atherosclerosis.^270^ AMPK activation also suppresses lipogenesis and enhances lipid disposal, including inhibition of sterol regulatory element-binding pathways, increased fatty-acid oxidation, and increased expression of hepatobiliary cholesterol transporters.^149^

AICAR increases LDL receptor mRNA and protein expression in hepatocytes through an ERK1/2-dependent, AMPK-independent mechanism and suppresses PPAR*γ* expression during monocyte-to-macrophage differentiation, thereby reducing the inflammatory response to oxidized LDL and saturated fatty acids (Fig. 5).^167^^,^^168^ These findings suggest potential effects on both the handling of circulating LDL and the biological consequences of lipoprotein modification in tissues.^344^ The combination of AICAR-mediated upregulation of the LDL receptor in hepatocytes and AMPK-mediated inhibition of HMG-CoA reductase and sterol regulatory element-binding protein 1c provides a mechanistic parallel to the complementary effects of statins and metformin on hepatic lipid handling.^167^^,^^345^ Given that sterol regulatory element-binding protein 1c drives hepatic de novo lipogenesis, its suppression may be particularly relevant to mixed dyslipidemia in metabolic dysfunction-associated steatotic liver disease, which frequently coexists with atherogenic dyslipidemia and accelerates cardiovascular risk.^346^

JAK/STAT inhibition decreases the expression of the scavenger receptors CD36 and CD204, reduces the uptake of oxidized LDL by macrophages, and increases ABCA1-mediated cholesterol efflux to apolipoprotein A-I, thereby promoting HDL formation (Fig. 6).^184^, ^185^, ^186^ Since dyslipidemia in insulin-resistant states often involves excess remnant lipoproteins, small, dense LDL, and impaired HDL function, these mechanisms may also be relevant to mixed dyslipidemias.^347^

HMGB1 inhibition has been shown to reduce serum lipid concentrations and atherosclerotic plaque burden in experimental models (Fig. 7).^225^ The relevance of HMGB1 as a target in dyslipidemia is supported by associations between its circulating levels and serum triglycerides and non-HDL cholesterol, as well as by studies showing that HMGB1-AGER signaling amplifies lipid-induced inflammation in macrophages and hepatocytes.^348^^,^^349^

Furthermore, the effects of methotrexate on the gut microbiota, particularly the reduction in *P copri* and *Bacteroides* (Fig. 8), may influence lipid metabolism, given the significant associations reported between these microbes and total cholesterol, LDL-cholesterol, and triglyceride concentrations in patients with coronary artery disease.^252^^,^^253^ Alterations in *P copri* and members of the *Bacteroides* genus are also associated with coronary heart disease-related gut dysbiosis and metabolic phenotypes relevant to bile acid/fibroblast growth factor 19 signaling and lipoprotein homeostasis.^350^^,^^351^

Taken together, these pathways suggest that methotrexate may beneficially modulate lipid trafficking and the biological toxicity of atherogenic lipoproteins by affecting cholesterol efflux, LDL receptor regulation, macrophage lipid uptake, inflammation-linked HDL dysfunction, and microbiota-associated metabolic signaling.

##### c Human studies

A secondary analysis of the Optimized Treatment Algorithm for Patients With Early Rheumatoid Arthritis trial, comparing the effects of combined treatment with the TNF-*α* inhibitor adalimumab and methotrexate (*n* = 86) versus methotrexate and placebo (*n* = 88), showed no significant differences in 1-year changes in the concentrations of total cholesterol, LDL-cholesterol, HDL-cholesterol, or triglycerides.^352^ An observational study of 262 patients with psoriasis investigated the impact of the rs11960458 polymorphism in annexin A6, a protein that regulates cholesterol homeostasis,^353^ on the effects of 12-week methotrexate treatment on the lipid profile. Methotrexate significantly reduced total cholesterol (*P* < .05), LDL-cholesterol (*P* < .05), apolipoprotein B (*P* < .001), and lipoprotein A (*P* < .001) concentrations in TC and CC genotype carriers of *rs11960458,* and apolipoprotein B (*P* = .04) in TT carriers. However, methotrexate also significantly reduced the concentrations of the atheroprotective subfractions HDL-cholesterol (*P* = .007) and apolipoprotein A1 (*P* = .04) in TC carriers. Moreover, it significantly reduced triglyceride concentrations only in CC carriers (*P* = .01).^354^ In another study of 288 patients with psoriasis, 136 with and 152 without concomitant psoriatic arthritis, 12-week treatment with methotrexate significantly lowered apolipoprotein B (*P* = .0003), total cholesterol (*P* = .0007), triglycerides (*P* = .04), HDL-cholesterol (*P* = .037), and lipoprotein A (*P* = .005) concentrations in patients with arthritis, and apolipoprotein B (*P* < .0001), total cholesterol (*P* < .0001), HDL-cholesterol (*P* = .011), LDL-cholesterol (*P* = .0001), and lipoprotein A (*P* < .0001) in patients without arthritis.^355^ In another study involving 35 patients with psoriasis, 12-week methotrexate treatment significantly reduced proprotein convertase subtilisin/kexin type 9 concentrations.^356^ This protein, which is critically involved in cholesterol homeostasis by binding to the LDL receptor in hepatocytes, is an established therapeutic target for cardiovascular prevention.^357^ In the previously described CIRT study, 8-month treatment with methotrexate significantly decreased LDL-cholesterol (*P* < .0001) and triglyceride (*P* = .005) concentrations compared with placebo.^280^

#### 5 Hypertension

##### a Pathophysiology

Arterial hypertension is generally the result of a complex interplay among renal, neurohumoral, vascular, and immune alterations that reset the pressure-natriuresis relationship and increase systemic vascular resistance.^358^ Sodium retention, often manifesting as salt sensitivity, promotes volume expansion and activation of the renin-angiotensin-aldosterone system. In turn, excess angiotensin II and aldosterone promote oxidative stress, inflammation, and fibrotic remodeling in the kidney parenchyma and vasculature.^359^ Excessive sympathetic activity leads to arterial vasoconstriction, renin release, and tubular sodium reabsorption, thereby further reinforcing renin-angiotensin-aldosterone system signaling and impairing baroreflex sensitivity.^358^ In resistance arteries, endothelial dysfunction, reduced NO synthesis, increased endothelin-1 activity, capillary rarefaction, and inward remodeling further elevate peripheral resistance. Concomitantly, large-artery stiffening amplifies pulse pressure and systolic load, thereby promoting left ventricular hypertrophy and increasing the risk of myocardial ischemia.^360^ Experimental and clinical evidence also supports the proposition that hypertension-mediated dysregulation of innate and adaptive immunity promotes excessive proinflammatory cytokine production and end-organ injury, particularly in the heart, brain, retina, and kidney.^361^

##### b Potential therapeutic effects of methotrexate targeting pathophysiological mechanisms

Several methotrexate targets are relevant to the pathophysiology of hypertension. Adenosine-mediated A_2A_ receptor activation induces vascular smooth muscle cell relaxation by opening voltage-gated and ATP-sensitive potassium channels, resulting in hyperpolarization and reduced peripheral vascular resistance.^101^ A_2A_ receptor stimulation also enhances eNOS activity in endothelial cells, although adenosine-mediated blood pressure-lowering effects may occur through eNOS-independent mechanisms (Fig. 3; Table 1).^102^^,^^362^ In addition to its vasodilatory effects on resistance arteries, adenosine influences blood pressure through central and renal mechanisms. A_2A_-mediated effects on the brainstem lower blood pressure by reducing central sympathetic drive, an effect distinct from peripheral vasodilation and potentially relevant to states of sympathetic overactivity.^363^^,^^364^ In the kidney, A_2A_ activation contributes to renal vasodilation and reduces renal vascular resistance, while attenuating tubuloglomerular feedback-mediated vasoconstriction via eNOS-dependent mechanisms. Through the renal A_2A_-epoxyeicosatrienoic acid pathway, these effects facilitate natriuresis and reduce blood pressure.^102^^,^^365^^,^^366^ Furthermore, adenosine suppresses renin release from juxtaglomerular cells predominantly through A_1_ receptor activation, thereby attenuating renin-angiotensin-aldosterone system-driven sodium retention and vasoconstriction.^367^^,^^368^ These multilevel effects on sympathetic tone, renal hemodynamics, and neurohormonal activation complement the direct vascular smooth muscle relaxation discussed above and suggest a broader antihypertensive profile for methotrexate-induced adenosine accumulation.

AMPK activation by methotrexate improves endothelium-dependent vasodilation and reduces inflammation in perivascular adipose tissue, a critical regulator of vascular tone (Fig. 4).^145^^,^^146^ AMPK phosphorylates eNOS at Ser1177 in a calcium-independent manner, directly increasing constitutive NO production in endothelial cells, an effect demonstrated by both AICAR and metformin.^369^, ^370^, ^371^ Because perivascular adipose dysfunction contributes to oxidative stress, altered paracrine signaling, and vascular stiffness, these observations provide an additional mechanistic rationale for the antihypertensive effects and are consistent with improvements in arterial stiffness reported in methotrexate-treated patients with rheumatoid arthritis.^372^^,^^373^ AMPK can also inhibit the Rho/Rho-kinase pathway in vascular smooth muscle cells, a signaling cascade that mediates sustained vasoconstriction, which is upregulated in several forms of hypertension.^374^

Inhibition of the JAK/STAT pathway prevents the overproduction of endothelin-1, a potent endogenous vasoconstrictor implicated in hypertension, by endothelial cells exposed to high glucose concentrations (Fig. 6).^187^^,^^188^ This is particularly relevant in patients with concomitant cardiometabolic disease, in whom endothelial inflammation and endothelin signaling often coexist.^375^ JAK/STAT signaling is also emerging as a contributor to hypertension-mediated renal injury. In angiotensin II-driven hypertension, renal cortical JAK2-STAT3 activation is associated with renal injury, and in renal tubular epithelial cells, angiotensin II-induced STAT3 activation promotes profibrotic responses; these pathways are attenuated by JAK2/STAT3 inhibition.^376^^,^^377^ Moreover, immune signaling involving T-cell IFN-gamma and IL-17A contributes to hypertension by promoting renal and vascular inflammation, immune cell infiltration, and disordered sodium handling.^378^, ^379^, ^380^ Because IFN-gamma signals through JAK/STAT pathways, these findings provide a plausible mechanistic link between methotrexate’s immunomodulatory effects and its blood pressure-lowering effects.

Additionally, HMGB1 inhibition may contribute to blood pressure reduction by attenuating vascular inflammation and smooth muscle cell proliferation (Fig. 7).^224^^,^^271^ HMGB1 contributes to hypertensive end-organ injury through distinct pathways. In the vasculature, extracellular HMGB1 can activate proremodeling signaling in vascular smooth muscle cells, including TLR4- and AGER-linked pathways, thereby promoting their proliferation and migration.^381^, ^382^, ^383^ In the kidney, HMGB1-TLR4 signaling in tubular epithelial cells drives tubulointerstitial inflammation and fibrosis, and circulating HMGB1 levels correlate with renal dysfunction.^384^, ^385^, ^386^ In the brain, HMGB1 signaling in the rostral ventrolateral medulla increases sympathetic nerve activity and elevates blood pressure, effects that are attenuated by blockade of the HMGB1/receptor for advanced glycation endproducts axis.^387^ Since hypertension-related target organ injury is increasingly understood to involve activation of innate and adaptive immunity, the broader anti-inflammatory and immunomodulatory actions of methotrexate may also be relevant to limiting vascular and renal damage.^361^ The ability of methotrexate to bind HMGB1 directly may therefore offer a plausible means of attenuating central, renal, and vascular mechanisms that sustain the hypertensive state and its complications.^220^

Therefore, methotrexate could exert antihypertensive effects by favorably influencing arterial stiffness, endothelial dysfunction, vascular inflammation, and target organ injury.

##### c Human studies

In a repeated cross-sectional study of 86 patients with stable rheumatoid arthritis, the use of methotrexate was associated with significantly lower systolic blood pressure (SBP; *P* = .006) and diastolic blood pressure (DBP; *P* = .001) compared with other csDMARDs, after adjusting for age, sex, body mass index, folic acid use, and disease activity.^372^ In further analyses, an increase in arterial stiffness at baseline was associated with a significant time-related increase in arterial blood pressure in rheumatoid arthritis patients not receiving methotrexate. However, methotrexate users showed no significant increase in arterial blood pressure over time.^388^ Another study examined administrative data to assess changes in arterial blood pressure among 21,916 patients with rheumatoid arthritis who started treatment with methotrexate, other csDMARDs such as leflunomide, sulfasalazine, or hydroxychloroquine, TNF-*α* inhibitors, or prednisone. A significantly higher proportion of patients on methotrexate achieved optimal blood pressure control at 6 months than those on other csDMARDs, TNF-*α* inhibitors, or prednisone.^389^

Two recent intervention studies have offered further insights into the potential impact of methotrexate on arterial blood pressure. A secondary analysis of the CIRT study compared the effects of low-dose methotrexate (*n* = 2391) versus placebo (*n* = 2395) on SBP and DBP in high-risk cardiovascular patients without autoimmune disease, with both treatment groups receiving folic acid (1 mg/d).^390^ Methotrexate treatment (median dose, 20 mg/wk) did not significantly reduce SBP or DBP compared with placebo over a mean follow-up of 26 months (ΔSBP = −0.75 mm Hg; 95% CI, −0.02 to −1.49; ΔDBP = −0.56 mm Hg; 95% CI, −0.12 to −1.01). Another study investigated the effects of a 6-month treatment with low-dose methotrexate (*n* = 31) or the csDMARD sulfasalazine (*n* = 31) on SBP, DBP, and the augmentation index (an indirect marker of arterial stiffness) in treatment-naïve patients with recently diagnosed rheumatoid arthritis.^391^ Compared with sulfasalazine, treatment with methotrexate was associated with a significant decrease in SBP (−7.4 mm Hg; 95% CI, −14.0 to −0.8; *P* = .03). However, there were no significant between-group differences in DBP or the augmentation index. The effects of methotrexate on SBP did not coincide with changes in disease activity (Disease Activity Score-28). In further analyses, the SNPs rs1801133 (methylenetetrahydrofolate reductase) and rs2231142 (ATP-binding cassette subfamily G member 2) had significant effects on changes in arterial blood pressure during methotrexate treatment, after adjusting for age, sex, and baseline blood pressure.

#### 6 Central adiposity and obesity

##### a Pathophysiology

Obesity is a chronic condition characterized by dysregulated energy homeostasis, in which genetic susceptibility and an obesogenic environment drive a positive energy balance. The expansion of visceral adipose tissue, involving both hypertrophy and hyperplasia, leads to local adipokine dysregulation, endoplasmic reticulum stress, mitochondrial dysfunction, hypoxia, and immune cell infiltration, resulting in a state of low-grade systemic inflammation that promotes insulin resistance and ectopic lipid deposition in several organs and tissues, particularly the liver, muscle, and myocardium.^392^ In parallel, obesity is associated with reduced thermogenic capacity and dysfunction of brown and beige adipose tissue.^393^ Central mechanisms favoring central adiposity and obesity include impaired hypothalamic sensing of leptin and nutrient signals. These alterations reinforce hyperphagia and reduce energy expenditure, progressively altering a wide range of autonomic and endocrine homeostatic mechanisms and leading to a wide range of complications.^394^ Gut-derived inflammatory signaling may further amplify adipose dysfunction and systemic metabolic injury.^395^

##### b Potential therapeutic effects of methotrexate targeting pathophysiological mechanisms

Several recently identified methotrexate targets are potentially relevant to the pathophysiology of obesity. The A_2B_ receptor, whose expression is negatively correlated with body mass index and age, plays a critical role in the interplay among adipose tissue, skeletal muscle, and aging. Its activation reduces oxidative stress, lipid peroxidation, and tissue senescence in brown adipose tissue, increases energy expenditure, and prevents diet-induced obesity in experimental models (Fig. 3; Table 1).^113^ These effects closely align with the inflammatory, mitochondrial, and thermogenic disturbances that characterize central adiposity.^392^ Because A_2B_ receptor expression is reduced in overweight individuals and is inversely related to aging-associated pathways in skeletal muscle, this pathway may also be relevant to the decline in metabolic elasticity associated with aging.^396^ A_2B_ receptor signaling also opposes the development of adipose tissue fibrosis, a pathological feature of chronic obesity that impairs adipocyte lipid storage capacity, promotes ectopic lipid deposition, and sustains low-grade inflammation through activation of hypoxia-inducible factor 1*α* and the formation of macrophage crown-like structures.^397^^,^^398^ A methotrexate-induced increase in extracellular adenosine may engage A_2B_ receptor-mediated pathways in these tissues, although the relative contribution of different adenosine receptor subtypes in the context of methotrexate therapy remains to be determined.

AMPK activation enhances lipid mobilization and oxidative disposal over storage through phosphorylation of adipose triglyceride lipase/desnutrin and hormone-sensitive lipase (Fig. 4).^104^^,^^141^^,^^399^ AMPK also suppresses the expression of proinflammatory adipokines, including leptin, resistin, and MCP-1, while upregulating the anti-inflammatory adipokine adiponectin, which itself activates AMPK in a positive feedback loop that reinforces insulin sensitization and anti-inflammatory signaling.^399^, ^400^, ^401^, ^402^ Adiponectin concentrations are reduced in central obesity and are inversely associated with visceral fat area, insulin resistance, and cardiovascular risk.^403^^,^^404^ By restoring the AMPK-adiponectin axis, methotrexate may therefore target a central node in the metabolic dysfunction that links visceral adiposity to cardiometabolic disease. These observations are consistent with the significant lipolytic effects of methotrexate reported in the visceral adipose tissue of diabetic mice.^151^

JAK/STAT signaling also contributes to obesity-driven inflammation and metabolic dysfunction (Fig. 6). Persistent activation of the hypothalamic leptin-JAK2-STAT3-suppressor of cytokine signaling 3 axis contributes to leptin resistance in obesity, thereby disrupting the central regulation of appetite and energy expenditure.^405^ Downregulation of leptin signaling, particularly of suppressor of cytokine signaling 3, improves hypothalamic leptin responsiveness and limits diet-induced obesity, providing a potential mechanistic link between the JAK/STAT inhibitory effects of methotrexate and central appetite regulation.^406^ In adipose tissue macrophages, STAT1-associated JAK/STAT signaling contributes to the proinflammatory macrophage polarization seen in visceral obesity, and interference with JAK/STAT signaling can shift macrophages toward a less inflammatory phenotype.^407^, ^408^, ^409^

HMGB1, which is excessively released from necrotic adipocytes and from those exposed to proinflammatory stimuli such as TNF-*α*, contributes to local and systemic inflammation in obesity and insulin resistance; methotrexate binding to HMGB1 may attenuate these effects (Fig. 7).^215^, ^216^, ^217^, ^218^, ^219^, ^220^, ^221^ Circulating HMGB1 concentrations correlate with body mass index and insulin resistance in obesity and may decline following major weight-loss interventions, including bariatric surgery, suggesting a reversible relationship between adiposity and HMGB1-mediated inflammation.^216^^,^^348^^,^^410^

The effects of methotrexate on gut microbiota composition, particularly the reduction in *Bacteroidetes* (Fig. 8), may also be relevant, as increased *Bacteroidetes* abundance in obesity leads to excess production of lipopolysaccharides and activation of proinflammatory TLR-4 and NF-*κ*B signaling pathways, as well as increased acetate production, which stimulates appetite through the parasympathetic nervous system.^245^^,^^249^^,^^250^

Collectively, these pathways suggest that methotrexate could influence the pathogenic consequences of central adiposity by targeting adipose inflammation, oxidative stress, impaired substrate oxidation, hypothalamic nutrient-sensing defects, and altered gut-immune signaling.

##### c Human studies

There is limited evidence regarding the effect of methotrexate on measures of central adiposity and obesity. Observational studies of patients with rheumatoid arthritis have reported relatively small increases in body mass index (∼0.2 kg/m^2^) after 12 months of treatment.^411^ This increase was similar to that observed with TNF-*α* inhibitors but lower than that seen with glucocorticoids (∼0.4 kg/m^2^). In other studies, long-term treatment with methotrexate, but not with TNF-*α* inhibitors or glucocorticoids, was independently associated with a lower risk of weight loss in rheumatoid arthritis.^412^

### B Age-related neurodegenerative diseases

#### 1 Alzheimer disease, vascular dementia, Lewy body dementia, and frontotemporal dementia

##### a Pathophysiology

Dementia comprises a range of syndromes primarily characterized by progressive cognitive impairment resulting from distinct yet frequently co-occurring brain pathologies. In Alzheimer disease, one of the main forms of dementia, a prolonged preclinical phase of amyloid-*β* accumulation is followed by pathogenic tau protein accumulation, synaptic failure, and neurodegeneration, with glial activation and immune-metabolic dysregulation.^413^ Vascular dementia reflects cumulative ischemic events in large and small vessels, including microinfarcts, microbleeds, and white matter disruption. Their occurrence leads to a disconnect between neuronal activity and cerebral blood flow, blood-brain barrier dysfunction, and chronic hypoperfusion, which together favor neurodegenerative pathways.^414^ Lewy body dementia is an *α*-synucleinopathy characterized by misfolded *α*-synuclein aggregates in cortico-limbic and brainstem circuits, resulting in cholinergic and dopaminergic deficits and clinically significant neuropsychiatric and behavioral fluctuations. The co-occurrence of Alzheimer pathology often accelerates cognitive and functional decline.^415^ Frontotemporal dementia results from selective degeneration of the frontotemporal neuronal network, driven by tauopathies or specific proteinopathies, and is accompanied by disturbances in RNA metabolism, proteostasis, and synaptic homeostasis.^416^ Prion diseases represent prototypic rapidly progressive dementias, driven by templated misfolding and the spread of the scrapie isoform of the prion protein, resulting in rapid neuronal injury.^417^ Across different forms of dementia, shared downstream mechanisms, including microglial priming, complement-mediated synapse loss, mitochondrial and lysosomal dysfunction, and impaired clearance of misfolded proteins, further promote neuronal vulnerability.^413^ In late life, mixed pathologies are common. For example, ischemic insults and coaggregating proteins often combine to reduce cognitive reserve and drive disease progression.^414^

##### b Potential therapeutic effects of methotrexate targeting pathophysiological mechanisms

Multiple mechanisms described in Section IV provide a framework for understanding how methotrexate could theoretically reduce the risk of various types of dementia. A_2A_ receptor antagonism enhances autophagy, exerts antioxidant effects via activation of the Kelch-like ECH-associated protein 1-nuclear factor erythroid 2-related factor 2 pathway, and improves synaptic function, memory, cerebral perfusion, and blood-brain barrier integrity in experimental models.^130^, ^131^, ^132^ Notably, combined A_1_ agonism and A_2A_ antagonism improved memory, increased IL-10 and postsynaptic density protein 95, and decreased TNF-*α* concentrations compared with either treatment alone in a lipopolysaccharide-induced model of Alzheimer disease (Fig. 3; Table 1).^114^ These effects are relevant not only to Alzheimer disease but also to vascular and mixed dementias, in which blood-brain barrier dysfunction, impaired perfusion, and neuroinflammation contribute to cognitive decline.^418^, ^419^, ^420^ Given the complex interplay between A_2A_ agonism and antagonism in the context of methotrexate-induced adenosine accumulation, the net effect on neurodegeneration likely depends on receptor subtype distribution, local adenosine concentrations, and the relative contributions of peripheral versus central adenosine signaling, which remains an important area for further investigation.

AMPK activation provides additional neuroprotective mechanisms relevant to multiple dementia subtypes. One of these mechanisms involves reduced amyloid-*β* generation and deposition by decreasing BACE1 abundance through suppression of mTOR, glycogen synthase kinase-3*β*, and NF-*κ*B signaling, while concurrently restraining mTORC1 activity, thereby reducing tau phosphorylation and upregulating autophagy to enhance the clearance of aggregation-prone proteins.^142^^,^^152^, ^153^, ^154^, ^155^, ^156^, ^157^ Furthermore, AMPK interacts with sirtuin 1 to suppress NF-*κ*B transcriptional activity and reduce neuroinflammation and oxidative injury.^141^^,^^158^^,^^159^ Although not yet directly investigated with methotrexate, several AMPK activators, including metformin, rosiglitazone, magnolol, and resveratrol, have demonstrated beneficial effects on cognitive function (Fig. 4).^160^, ^161^, ^162^, ^163^ Furthermore, AMPK-peroxisome proliferator-activated receptor *γ* coactivator 1-*α* signaling supports mitochondrial biogenesis and oxidative metabolism in neurons, processes that are compromised in both Alzheimer disease and vascular dementia.^421^^,^^422^ Mitochondrial dysfunction in neurodegenerative disorders promotes bioenergetic failure, oxidative stress, and activation of apoptotic pathways, all of which may be mitigated by restoring AMPK-proliferator-activated receptor *γ* coactivator 1-*α* signaling.^423^ AMPK activation has also been linked to improved blood-brain barrier integrity, including the preservation of tight junction proteins such as claudin-5 and occludin in experimental models; mechanisms that are directly relevant to vascular dementia and the cerebrovascular component of mixed dementias.^424^^,^^425^

JAK/STAT signaling, particularly the JAK2-STAT3 axis, plays a central role in reactive astrogliosis and glial neuroinflammation, which are prominent features of Alzheimer disease.^426^ Amyloid-*β* activates STAT3-dependent astrocyte reactivity, and JAK/STAT3 signaling is now recognized as a common inducer of the neurotoxic reactive astrocyte phenotype.^427^^,^^428^ Consistent with this, astrocyte-specific STAT3 deletion in an Alzheimer disease mouse model reduced gliosis, lowered amyloid burden, improved plaque-associated microglial handling, and ameliorated network and cognitive dysfunction.^429^ JAK/STAT inhibition enhances cognitive and behavioral function in experimental models (Fig. 6).^196^^,^^197^ The natural flavonoid and JAK/STAT inhibitor resveratrol significantly reduced cognitive impairment following cerebral ischemia-reperfusion injury in rats, along with reduced anxiety-like behavior, hippocampal neuronal loss, antioxidant and anti-inflammatory effects, and suppression of cell apoptotic pathways.^196^ In a murine model of human immunodeficiency virus-related neurocognitive disorder, baricitinib penetrated the blood-brain barrier, improved cognitive and behavioral abnormalities, and reduced neuroinflammation markers.^197^

HMGB1, whose concentrations are significantly elevated in the cerebrospinal fluid of patients with Alzheimer disease, promotes amyloid-*β* accumulation and drives tau hyperactivity and aggregation, leading to cognitive impairment and frontotemporal dementia.^208^^,^^430^^,^^431^ Treatment with HMGB1 inhibitors, such as ethyl pyruvate and glycyrrhizic acid, significantly reduces APOE4-driven gliosis and neurodegeneration, as indicated by increased hippocampal volume (Fig. 7).^227^

Furthermore, the effects of methotrexate on gut microbiota composition may be relevant, given the significant associations between *Bacteroidetes* and the risk of Alzheimer disease, mediated by excess production of lipopolysaccharides and tryptophan metabolites, and the consequent modulation of microglial maturation, immune signaling, and neuroinflammation through systemic, vagal, and metabolic pathways.^432^, ^433^, ^434^, ^435^

Overall, the potential beneficial effects of methotrexate in Alzheimer disease, vascular dementia, and mixed dementia are biologically plausible because it targets protein aggregation, autophagy failure, neuroinflammation, oxidative stress, cerebrovascular dysfunction, blood-brain barrier impairment, and gut-brain communication.

##### c Human studies

A study using data from an insurance database in the USA identified 957 rheumatoid patients with dementia not otherwise specified, and another 957 without dementia.^436^ Univariate analysis showed that both <395-day and ≥395-day methotrexate treatment were associated with a significantly higher risk of dementia not otherwise specified compared with no csDMARD use (OR, 1.70; 95% CI, 1.29–2.24; and OR, 1.58; 95% CI, 1.20–2.07, respectively). Similar associations were observed with other csDMARDs, such as hydroxychloroquine and sulfasalazine. By contrast, in another study of patients with rheumatoid arthritis using the UK Clinical Practice Research Datalink, 3876 csDMARD users were propensity score-matched to 1938 nonusers.^437^ A significant reduction in the 15-year risk of dementia not otherwise specified was observed among csDMARD users (HR, 0.60; 95% CI, 0.42–0.85), particularly those receiving methotrexate (HR, 0.52; 95% CI, 0.34–0.82). Similarly, in a retrospective matched case-control study involving rheumatoid arthritis patients with (*n* = 486) and without (*n* = 641) dementia not otherwise specified, the use of methotrexate but not sulfasalazine was associated with a significantly lower risk of dementia not otherwise specified after adjusting for age, body mass index, stroke, and myocardial infarction (aOR, 0.72; 95% CI, 0.52–0.98).^438^ In further analyses, the effect of methotrexate was especially apparent at longer treatment durations exceeding 4 years (aOR, 0.38; 95% CI, 0.18–0.84). Another retrospective case-control study using electronic health records of patients with rheumatoid arthritis and psoriasis reported that treatment with methotrexate was associated with a significantly reduced risk of Alzheimer disease (aOR, 0.64; 95% CI, 0.61–0.68) and dementia in general (aOR, 0.58; 95% CI, 0.56–0.60).^439^ By contrast, a retrospective population-based cohort study of 886 patients with rheumatoid arthritis reported no significant association between methotrexate treatment and incident dementia not otherwise specified after adjusting for age, sex, year of rheumatoid arthritis incidence, smoking, obesity, hypertension, diabetes mellitus, hyperlipidemia, and cardiovascular disease (HR, 1.17; 95% CI, 0.72–1.88).^440^

A recent systematic review and meta-analysis of 14 studies (5 on methotrexate) involving 940,442 patients with rheumatoid arthritis found that methotrexate use was associated with a lower, though not significant, risk of dementia not otherwise specified (RR, 0.78; 95% CI, 0.54–1.12).^441^ By comparison, the RR of hydroxychloroquine was 0.95 (95% CI, 0.63–1.44), whereas sulfasalazine was associated with an increased risk of dementia not otherwise specified (RR, 1.27; 95% CI, 1.06–1.50). The use of TNF-*α* inhibitors was associated with a significantly lower risk of dementia not otherwise specified. The heterogeneity in studies investigating TNF-*α* inhibitors (I^2^ = 0.0%–34.2%) was markedly lower than that observed with methotrexate (I^2^ = 91.3%), hydroxychloroquine (I^2^ = 81.3%), and sulfasalazine (I^2^ = 42.6%).

A Mendelian randomization study identified 127 potential immune system and blood-brain barrier biomarkers as risk factors for dementia not otherwise specified.^442^ These biomarkers were then examined in a phenome-wide analysis using Mendelian randomization-based polygenic risk scores in a cohort of 339,233 individuals, revealing a shared genetic background between autoimmune diseases and dementia not otherwise specified.^442^ In inverse-weighted probability analyses simulating randomized controlled drug trials using observational data, treatment with methotrexate was associated with a significant reduction in the risk of Alzheimer disease compared with no treatment (HR, 0.64; 95% CI, 0.49–0.88). Another 2-sample Mendelian randomization study using summary statistics from genome-wide association studies involving 462,933 individuals (2454 cases and 460,479 controls) reported a protective effect of methotrexate use on the risk of dementia not otherwise specified (OR, 0.476; 95% CI, 0.362–0.626).^443^

#### 2 Parkinson disease

##### a Pathophysiology

Parkinson disease is a progressive synucleinopathy defined pathologically by misfolded *α*-synuclein aggregates (Lewy pathology) and the selective vulnerability of nigrostriatal dopaminergic neurons. These processes are primarily responsible for basal ganglia circuit dysfunction and clinical motor manifestations, such as tremor, bradykinesia, and rigidity.^444^ Additional pathophysiological mechanisms, including impaired proteostasis (ubiquitin-proteasome and autophagy-lysosome systems), mitochondrial bioenergetic failure, oxidative stress, and disturbed Ca^2+^ homeostasis, amplify *α*-synuclein aggregation and neuronal injury.^445^ Pathogenic *α*-synuclein can propagate between neurons and glia, triggering maladaptive microglial and astroglial activation and a feed-forward neuroinflammatory environment that accelerates dopaminergic degeneration.^446^ Abnormal kinase signaling, including pathways involving LRRK2, further modulates inflammatory and degenerative responses.^447^ Importantly, neurodegeneration extends beyond the substantia nigra to several monoaminergic, cholinergic, and autonomic networks, contributing to nonmotor features and later cognitive and autonomic impairment.^448^

##### b Potential therapeutic effects of methotrexate targeting pathophysiological mechanisms

Multiple methotrexate targets are directly relevant to the pathophysiology of Parkinson disease. A_2A_ receptors, predominantly localized in the basal ganglia, regulate numerous neurotransmitters and colocalize with dopamine D_2_ receptors in the striatum, where they form heteromeric complexes with antagonistic interactions. A_2A_ receptor activation reduces D_2_ receptor signaling efficacy, whereas A_2A_ receptor blockade enhances dopaminergic transmission and improves motor function, even in the setting of dopaminergic denervation.^449^^,^^450^ This receptor-level interaction supports the results of studies with the A_2A_ antagonists preladenant and istradefylline, which report reversal of motor function impairment, neuroprotective effects, attenuation of proinflammatory markers, including TNF-*α*, neuronal NO synthase, and inducible NO synthase, and the preservation of anti-inflammatory markers such as TGF-*β*1 and IL-4 in experimental models and in patients with Parkinson disease (Fig. 3; Table 1).^116^, ^117^, ^118^, ^119^, ^120^ Given the central role of basal ganglia circuitry dysfunction in Parkinson disease, these findings provide a particularly strong mechanistic link between adenosine signaling and clinical manifestations.^451^

AMPK activation reduces 1-methyl-4-phenylpyridinium-positive–induced cell loss and exerts protective effects in Parkinson disease models, including increased autophagy, reduced microglial activation, reduced proinflammatory cytokine expression, decreased reactive oxygen species production, and attenuated mitochondrial dysfunction (Fig. 4).^165^^,^^166^ AMPK phosphorylates the mitochondrial fission factor, facilitating mitochondrial fragmentation and mitophagy, thereby supporting the removal of dysfunctional mitochondria from neurons that are especially vulnerable to bioenergetic stress.^452^^,^^453^ AMPK activation can also engage the peroxisome proliferator-activated receptor-*γ* coactivator 1-*α*-mitochondrial transcription factor A mitochondrial biogenesis axis, potentially helping to replenish the mitochondrial pool in neurons exposed to chronic oxidative stress.^454^ These effects are relevant to key disease mechanisms, including impaired proteostasis, oxidative stress, and bioenergetic failure.^455^ The AMPK-independent downregulation of LRRK2 expression by AICAR rescues dopaminergic neurodegeneration and reduces neuroinflammatory markers, such as CD68 (Fig. 5).^170^ This is particularly important given the contribution of abnormal kinase signaling to the pathogenesis of Parkinson disease and other neurodegenerative disorders.^456^ LRRK2 hyperactivation disrupts vesicular trafficking, perturbs Ras-associated binding proteins and GTPase-dependent endolysosomal pathways, promotes *α*-synuclein pathology, and amplifies inflammatory responses.^457^

JAK/STAT signaling also contributes to the propagation of *α*-synuclein pathology, a central feature of Parkinson disease. Extracellular *α*-synuclein aggregates activate microglial inflammatory signaling, including JAK/STAT- and STAT1-associated pathways, thereby triggering the release of proinflammatory mediators that create a neurotoxic milieu.^198^ In astrocytes, *α*-synuclein uptake promotes reactive phenotypic conversion via pathways including JAK/STAT3 signaling, thereby compromising trophic support and exacerbating neuronal vulnerability in the substantia nigra.^458^ The JAK1/2 inhibitor AZD1480 (Fig. 6) suppresses neuroinflammatory manifestations, including microglial activation, macrophage and CD4+ T-cell infiltration, and proinflammatory chemokine production, by inhibiting *α*-synuclein, thereby preventing dopaminergic neuron degeneration.^198^^,^^199^

Serum HMGB1 concentrations are significantly elevated in patients with Parkinson disease and correlate with disease progression and TLR4 expression.^459^ Extracellular HMGB1 can activate microglia and astrocytes via TLR4- and AGER-mediated pathways, thereby establishing a self-amplifying inflammatory loop within the substantia nigra.^459^ Furthermore, HMGB1 has been shown to perturb autophagic processing in dopaminergic cells through interactions with the beclin-1/vacuolar protein sorting 34 complex-associated machinery, thereby impairing the clearance of *α*-synuclein aggregates and promoting their intracellular accumulation.^460^ Treatment with anti-HMGB1 antibodies partially prevents dopaminergic neuron death and reduces AGER and TNF-*α* concentrations in murine models.^461^ Glycyrrhizic acid increased dopaminergic neuronal length, reduced apoptosis, and improved locomotor function in a zebrafish model.^228^ These effects were associated with reduced expression of proinflammatory genes, including *HMGB1a, tlr4b, nfκb, il1β, and il6* (Fig. 7).^228^ The direct binding of methotrexate to HMGB1 may therefore influence both extracellular inflammatory signaling and intracellular protein quality-control pathways relevant to Parkinsonian neurodegeneration.^220^^,^^221^

Gut microbiota modulation provides another plausible mechanism, as dysbiosis is increasingly implicated in the inflammatory and neurodegenerative processes underlying Parkinson disease.^462^ Furthermore, gastrointestinal dysfunction, including constipation and altered intestinal barrier function, often precedes motor symptoms by years and is consistent with the hypothesis that *α*-synuclein pathology may begin in the enteric nervous system and spread rostrally via connected pathways, including the vagus nerve.^463^ Methotrexate-induced modulation of gut microbiota composition may therefore influence enteric inflammatory signaling and other gut-brain axis mechanisms relevant to Parkinson disease pathogenesis.

Taken together, methotrexate could theoretically influence Parkinson disease through complementary effects on proteostasis, mitochondrial dysfunction, neuroimmune activation, abnormal kinase signaling, neuron-glia signaling, basal ganglia neurotransmission, and gut-brain communication.

##### c Human studies

A nationwide registry-based, nested, case-control study involving 22,189 patients with a confirmed clinical diagnosis of Parkinson disease identified 318 patients with concurrent rheumatoid arthritis and 1571 matched controls without the condition.^464^ The use of methotrexate was not significantly associated with Parkinson disease (aOR, 1.18; 95% CI, 0.91–1.53). Similarly, no significant associations were observed with sulfasalazine, gold compounds, other immunosuppressants, or corticosteroids. In contrast, the use of hydroxychloroquine was negatively associated with Parkinson disease. Another study, using the same nationwide registry, employed a systematic screening approach to identify drugs with potentially protective effects against Parkinson disease.^465^ The use of methotrexate was associated with a nonsignificant trend toward a decreased risk of Parkinson disease (OR, 0.51; 95% CI, 0.24–1.1; *P* = .085). More recently, a study assessed the association between methotrexate and Parkinson disease using a 2-sample Mendelian randomization methodology.^466^ An inverse-variance weighted approach showed that methotrexate use was significantly associated with a reduced risk of developing Parkinson disease (OR, 4.78E-11; 95% CI, 1.06E-19–2.16E-02; *P* = 1.94E-08).

### C Overall evaluation of the existing evidence from human studies

A significantly larger number of observational studies have examined the association between low-dose methotrexate treatment and the risk of age-related cardiometabolic diseases than neurodegenerative diseases. Notwithstanding this issue, virtually all studies have been observational and conducted on patients with autoimmune disorders, mainly rheumatoid arthritis. Furthermore, systematic reviews and meta-analyses examining specific endpoints, such as cardiovascular events, diabetes, and dementia not otherwise specified, have generally reported high heterogeneity (I^2^ > 75%), indicating substantial clinical and/or methodological differences across studies. This heterogeneity can render pooled estimates potentially unreliable and warrants careful interpretation. Given confounding by indication and related “channeling” processes, in which treatment allocation is driven by clinical factors that also predict outcomes, the inverse associations observed between low-dose methotrexate and age-related cardiometabolic and neurodegenerative diseases in these studies must be interpreted with caution.^467^ Methotrexate users may differ from nonusers in baseline disease activity, disease duration, frailty, renal and/or hepatic function, and treatment contraindications. Such factors can bias estimates toward apparent cardiometabolic and neuroprotective effects if higher-risk patients preferentially avoid or discontinue methotrexate early. Residual confounding by disease severity is particularly problematic because routine databases incompletely capture inflammatory burden (eg, Disease Activity Score-28/C-reactive protein trajectories), cumulative glucocorticoid exposure, and functional status, even when propensity score methods are used.^468^ Differential healthcare utilization (eg, more intensive monitoring, earlier detection of cardiometabolic and neurodegenerative diseases, and competing-risk survival) can further distort associations.^467^

#### 1 Age-related cardiometabolic diseases and risk factors

Overall, methotrexate treatment is associated with a decreased risk of atherosclerotic vascular disease and its principal manifestations, such as myocardial infarction, stroke, and heart failure, a reduced risk of diabetes mellitus, and beneficial effects on blood pressure control. In contrast, evidence regarding the impact of methotrexate on obesity and dyslipidemia is limited and uncertain (Fig. 9).

**Fig. 9:**
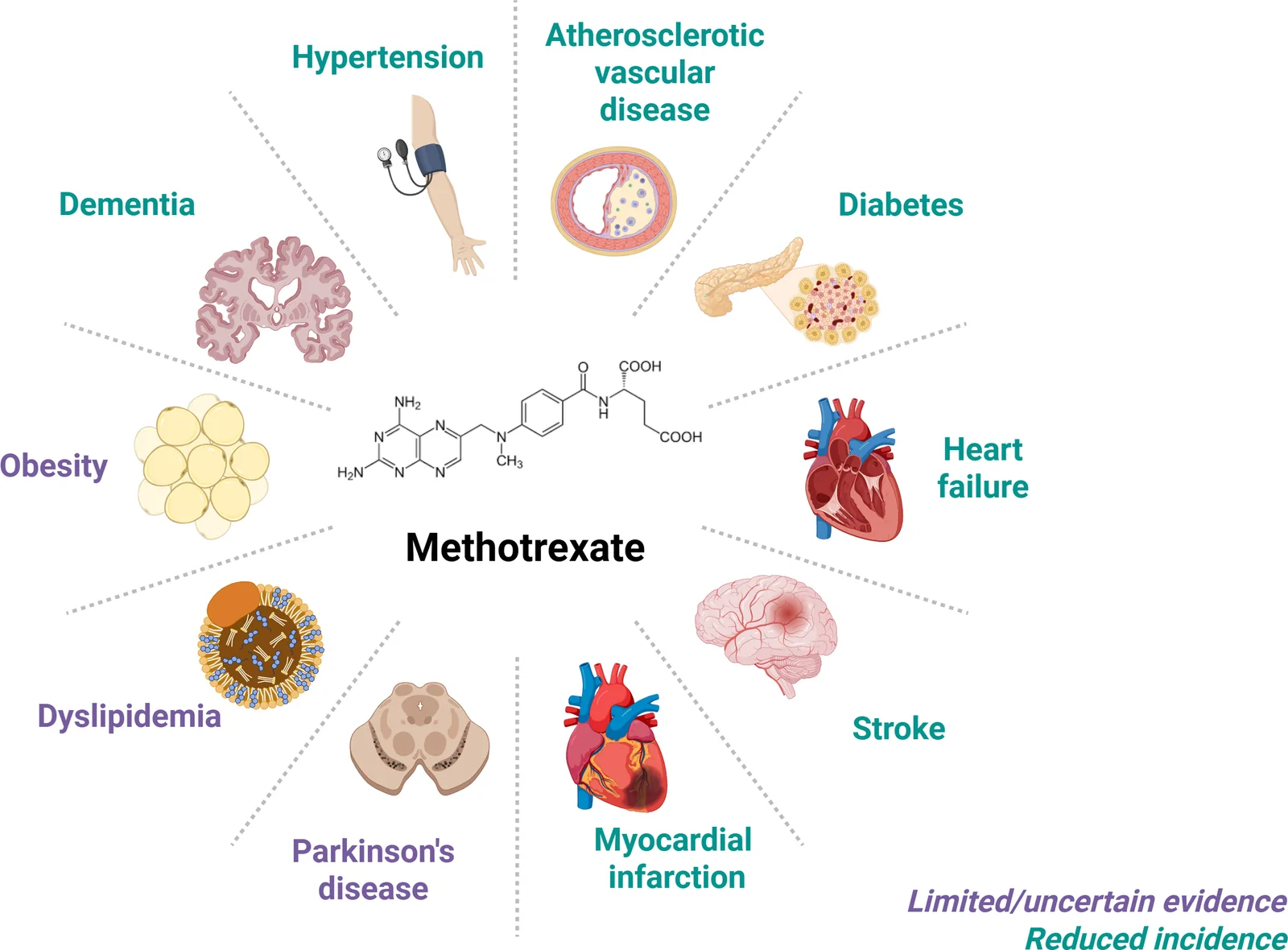
Summary of the evidence regarding the association between methotrexate and age-related cardiometabolic and neurodegenerative diseases.

The mixed effects observed across various lipid subfractions should also be considered in light of the complex interplay between lipids and inflammation in rheumatoid arthritis. Specifically, excess inflammation in rheumatoid arthritis has been shown to reduce total cholesterol and specific proatherogenic subfractions, such as LDL-cholesterol.^469^ This phenomenon, also known as the “lipid paradox,” may explain the increased risk of cardiovascular morbidity and mortality in patients with rheumatoid arthritis who have low LDL- or VLDL-cholesterol concentrations, indicating a particularly high inflammatory state.^470^ As part of the “lipid paradox,” anti-inflammatory treatment with methotrexate and other DMARDs has been shown to increase total cholesterol and LDL-cholesterol concentrations in patients with rheumatoid arthritis.^471^ However, it is also worth noting that methotrexate treatment significantly decreased LDL-cholesterol and triglyceride concentrations in patients without autoimmune disease and with a relatively low inflammatory burden.^280^

The only randomized controlled study investigating the effects of low-dose methotrexate on cardiovascular outcomes, specifically in participants without autoimmune diseases, the CIRT study, failed to show significant protective effects on atherosclerosis and blood pressure.^280^ Pending the results of further observational and intervention trials, an important feature of the CIRT study was that both the methotrexate and placebo groups received vitamin B folic acid treatment (1 mg/d). Previous studies have shown that folic acid exerts atheroprotective effects by lowering the highly reactive amino acid homocysteine,^472^ enhancing endothelial function,^473^ and reducing arterial stiffness and blood pressure.^474^^,^^475^ Such effects may explain the reduced risk of cardiovascular events observed with vitamin B in some intervention studies.^476^^,^^477^ Further research is necessary to determine whether the use of folic acid in the placebo group of the CIRT study may have reduced the potential atheroprotective effects of methotrexate, and whether these effects vary between patients with and without autoimmune conditions and across different levels of inflammatory burden.^478^ In this context, the median baseline CRP concentrations of CIRT participants (methotrexate group: 1.53 mg/L; placebo group: 1.50 mg/L)^280^ were considerably lower than those reported in studies showing a blood pressure-lowering effect of methotrexate in rheumatoid arthritis (methotrexate group (mean ± SD): 4.0 ± 3.6 mg/L; sulfasalazine group (mean ± SD): 7.0 ± 7.4 mg/L).^391^

#### 2 Age-related neurodegenerative diseases

Low-dose methotrexate is associated with a neutral or reduced risk of dementia, primarily investigated by assessing different types of dementia as a combined outcome in patients with autoimmune diseases (Fig. 9). There is limited evidence regarding the association between methotrexate treatment and Parkinson disease.

There is an established link between autoimmunity, inflammation, atherosclerosis, diabetes mellitus, and different types of dementia.^479^, ^480^, ^481^, ^482^, ^483^ Future research should investigate whether the potential neuroprotective effects of methotrexate are mediated by reductions in systemic inflammation and atherosclerotic burden, improvements in glycemic control, and/or a direct impact of methotrexate on the central nervous system.

## VI General considerations and future directions

The established pathophysiological roles of dysregulated immunity, inflammation, and redox signaling pathways in age-related cardiometabolic and neurodegenerative diseases continue to expand the range of candidate therapeutic targets. In parallel with the development of pathway-selective biological agents, such as the monoclonal antibody targeting IL-1*β*, canakinumab, and the repurposing of anti-inflammatory drugs, such as colchicine, for vascular protection,^40^^,^^54^ greater attention is now being directed toward approved agents with broader pleiotropic effects. In this context, the csDMARD methotrexate is particularly attractive because, unlike drugs that target a single cytokine or receptor, it acts on interconnected pathways that regulate inflammatory signaling, cellular bioenergetics, vascular homeostasis, immune cell activation, and potentially gut microbiota-host interactions. The close interplay among inflammaging, immunosenescence, metabolic dysfunction, cellular senescence, and tissue-specific stress responses further strengthens the rationale for investigating whether low-dose methotrexate may have disease-modifying effects beyond classical autoimmune indications.

The available human evidence remains strongest in patients with autoimmune disorders, particularly rheumatoid arthritis, in whom low-dose methotrexate has been associated with a reduced risk of atherosclerotic vascular disease and its principal manifestations, hypertension, heart failure, diabetes mellitus, and, in some studies, dementia. By contrast, evidence regarding dyslipidemia, obesity, and Parkinson disease remains limited. Furthermore, the neutral findings of the CIRT trial underscore the risk of extrapolating findings from autoimmune and inflammatory disease populations to participants without autoimmune disease and with a relatively low burden of systemic inflammation.^280^^,^^390^ This discrepancy is likely related to a range of factors, including differences in inflammatory burden, background folic acid exposure, concomitant therapies, disease stage, exposure duration, and endpoint selection. In addition, observational studies of rheumatoid arthritis and other autoimmune disorders remain vulnerable to channeling bias, residual confounding by disease severity and glucocorticoid exposure, and survivor effects, even when using advanced adjustment methods.^467^^,^^468^ Therefore, future epidemiological studies should place greater emphasis on disease activity trajectories, cumulative inflammatory burden, and time-varying exposure analyses, while future intervention studies should preferentially target individuals with clear evidence of residual inflammatory risk and a biologically plausible rationale for treatment response.

A major strength of methotrexate, as highlighted throughout this review, is its ability to target several processes directly relevant to the pathophysiology of age-related cardiometabolic and neurodegenerative diseases. The adenosine axis currently offers the most robust mechanistic framework. In addition to its well-established anti-inflammatory effects, adenosine signaling can promote cholesterol efflux, reduce foam cell transformation, support endothelial vasomotor function, modulate glucose homeostasis, and influence synaptic and cerebrovascular physiology. By contrast, evidence for AMPK activation is particularly strong in preclinical studies of vascular dysfunction, atherosclerosis, insulin resistance, and defective autophagy, whereas the AMPK-independent effects of AICAR remain promising but are more exploratory. Inhibition of JAK/STAT and HMGB1 is biologically compelling because it targets pathways involved in inflammaging, fibrosis, endothelial activation, cellular senescence, and neuroinflammation. However, they require more direct validation in methotrexate-treated experimental systems and in humans. Similarly, microbiome-mediated effects are increasingly plausible not only because of their potential contributions to systemic inflammation, cardiometabolic dysfunction, and neurodegeneration, but also because the gut microbiota can influence methotrexate pharmacokinetics, pharmacodynamics, and treatment response. Together, these observations support the proposition that methotrexate should not be considered solely as an antiproliferative or anti-inflammatory agent, but rather as a multitarget modulator of vascular, metabolic, and neuroimmune homeostasis.

Another important observation from recent mechanistic studies is that the methotrexate concentrations required to modulate several newly identified targets are often similar to those measured during low-dose treatment in clinical practice, supporting the translational relevance of these findings.^129^ This, in turn, strongly argues for incorporating target-engagement biomarkers into future studies by integrating established pharmacological markers, such as intracellular methotrexate polyglutamates, with pharmacogenetic determinants of transport, intracellular retention, folate metabolism, and adenosine signaling.^123^, ^124^, ^125^ Such an approach may help identify patient subgroups more likely to exhibit favorable effects on vascular inflammation, glucose metabolism, or neurodegenerative trajectories. Furthermore, it may clarify whether treatment failure in some individuals reflects inadequate exposure, insufficient intracellular polyglutamation, an unfavorable genetic background, or disease biology dominated by pathways that are less responsive to methotrexate.

Future clinical studies should therefore move beyond simple disease-based categorization by including, in age-related cardiometabolic diseases, participants with persistent residual inflammatory risk despite optimal standard therapy; evidence of endothelial dysfunction or arterial inflammation; metabolic syndrome with increased inflammatory burden; or autoimmune disease phenotypes associated with accelerated vascular aging. In age-related neurodegenerative diseases, early or prodromal stages, mixed vascular-neurodegenerative phenotypes, and patients with biomarker evidence of neuroinflammation or concomitant cardiometabolic dysfunction may represent more appropriate targets than advanced disease, in which irreversible neuronal loss is likely to limit pharmacological benefit. Similarly, endpoints should align with the expected mechanism and time course of action, combining hard clinical outcomes with intermediate measures such as inflammatory biomarkers, vascular imaging, endothelial function, insulin sensitivity, body composition metrics, cognitive trajectories, and, where feasible, blood or cerebrospinal fluid biomarkers of neurodegeneration and neuroinflammation.

The possibility that methotrexate may influence neurodegenerative disease both indirectly and directly deserves particular attention. On the one hand, reduced systemic inflammation, attenuation of atherosclerotic burden, improved endothelial function, and better metabolic control could reduce the risk of all-cause dementia, Alzheimer disease, vascular dementia, and Parkinson disease. On the other hand, several experimental observations suggest potential direct effects on autophagy, amyloid-*β* clearance, microglial activation, synaptic integrity, and neuronal survival. However, the penetration of methotrexate into the central nervous system is limited, with approximately 5% of the free drug in plasma^484^ due to active efflux transporters located at the blood-brain barrier. Relevant transporters include ATP-binding cassette subfamily G member 2 and, potentially, organic anion transporters such as organic anion-transporting polypeptide 1A2 and organic anion transporter 3.^485^ Future work should therefore address whether systemic inflammatory states, blood-brain barrier dysfunction, or new delivery platforms can meaningfully increase central nervous system exposure, and whether such changes translate into measurable neuroprotective effects.^486^^,^^487^

The gut microbiota represents another particularly promising area for future research. Recent findings that pretreatment microbial composition may predict methotrexate response, together with evidence that specific microbes can alter methotrexate metabolism and systemic exposure, suggest that host-microbe interactions may be central to the variability in both efficacy and toxicity. This has at least 3 implications. First, microbiome profiling may help identify patients most likely to benefit from methotrexate-based repurposing strategies. Second, microbial modulation, whether dietary, probiotic, or otherwise, may eventually enhance treatment response or tolerability. Third, future studies should consider the microbiota not only as a downstream mediator of methotrexate effects but also as a determinant of drug disposition and target engagement.

From a practical perspective, potential advantages of using low-dose methotrexate in older adults include its dosing schedule, alternative routes of administration, and favorable safety profile.^488^ Weekly dosing is unlikely to add significant complexity to the daily polypharmacy burden in this population and may improve treatment adherence.^489^^,^^490^ The availability of subcutaneous formulations offers a valuable option for optimizing bioavailability, pharmacokinetic predictability, and clinical efficacy.^73^^,^^491^ Furthermore, when proper dosing and clinical monitoring are followed, the known hepatic, gastrointestinal, and hematological side effects associated with low-dose methotrexate are infrequent and rarely severe. In a naturalistic study of 673 patients with inflammatory arthritis, primarily rheumatoid arthritis, 74% remained on oral methotrexate after 5 years.^492^ Methotrexate was discontinued in 10.8% of patients due to gastrointestinal side effects, 5.5% due to abnormal liver function tests, and another 5.5% because of hematological abnormalities. Severe life-threatening side effects were identified in 1.8% of patients.^492^ A systematic review of 88 relevant articles found that long-term, low-dose methotrexate treatment in patients with rheumatoid arthritis was not significantly associated with an increased risk of serious infections.^493^ The prevalence of abnormal liver test results was approximately 13%, with 10.5% of patients stopping treatment, including 3.7% due to hepatotoxicity. Furthermore, in the CIRT study, which included participants without autoimmune conditions, the total incidence of serious adverse events did not differ significantly between the methotrexate and placebo groups (*P* = .52).^280^ In comparison, observational and intervention trials with statins have reported an incidence of statin-associated muscle symptoms ranging from 4.9% to 30%, and epidemiological studies have reported that 17.5% of patients discontinued these agents due to a statin-related event.^494^, ^495^, ^496^

In summary, the available evidence supports further investigation into the repurposing of low-dose methotrexate for selected age-related cardiometabolic and neurodegenerative diseases. The rationale is based on the combination of clinically relevant target engagement, multipathway modulation, longstanding human experience, and plausible links to key mechanisms of aging-related tissue dysfunction. Future studies should consider mechanistic recruitment stratification (eg, participants with persistent residual inflammatory risk, endothelial dysfunction, arterial inflammation, and metabolic syndrome with increased inflammatory burden), a biomarker-guided trial design, assessment of tissue-specific exposure and target engagement, and possible integration with existing cardioprotective and neuroprotective therapies. This approach is likely to determine whether methotrexate has a genuine therapeutic role in age-related cardiometabolic and neurodegenerative diseases, and, if so, in which biological and clinical contexts that role is most likely to emerge.

## Conflict of interest

Arduino A. Mangoni received funding from medac GmbH to conduct an investigator-initiated clinical trial on the effects of methotrexate on blood pressure and arterial function in patients with rheumatoid arthritis. All other authors declare no conflicts of interest.
